# Supplementary material for: Cohort profile: InfCareHIV, a prospective registry-based cohort study of people with diagnosed HIV in Sweden
Source: BMJ Open. 2023 Mar 17;13(3):e069688. doi: 10.1136/bmjopen-2022-069688 (PMC10030896; doi:10.1136/bmjopen-2022-069688)
Supplement: Supplementary data [file bmjopen-2022-069688supp001.pdf]

Supplementary Table 1
Epidemiology of the InfCareHIV cohort

**Year of HIV diagnosis**	**<1987**	**1987-1996**	**1997-2006**	**2007-2016**	**2017-2022**	**unknown**	**All**
**All**	1264 (9.7)	2602 (20.0)	3076 (23.6)	4438 (34.1)	1320 (10.1)	324 (2.5)	13029
**Sex**							
Female	163 (12.9)	729 (28.0)	1225 (39.8)	1685 (38.0)	433 (33.1)	129 (42.3)	4364 (33.5)
**Birth region**							
Sweden	786 (62.2)	1153 (44.3)	1060 (34.5)	1131 (25.5)	362 (27.4)	36 (10.9)	4528 (34.7)
Western Europe except Sweden	128 (10.1)	225 (8.7)	193 (6.3)	233 (5.2)	53 (4.0)	10 (3.0)	842 (6.5)
Eastern Europe and Central Asia	22 (1.7)	60 (2.3)	169 (5.5)	391 (8.8)	152 (11.5)	39 (11.8)	833 (6.5)
Asia and Pacific	6 (0.5)	77 (3.0)	313 (10.2)	470 (10.6)	149 (11.3)	29 (8.8)	1044 (8.0)
Middle East and North Africa	6 (0.5)	33 (1.3)	53 (1.7)	149 (3.4)	63 (4.8)	8 (2.4)	312 (2.4)
Sub-Saharan Africa	26 (2.1)	632 (24.3)	1125 (36.6)	1775 (40.0)	421 (31.9)	96 (29.2)	4 075 (31.3)
Latin America and the Caribbean	19 (1.5)	92 (3.5)	115 (3.7)	228 (5.1)	85 (6.4)	10 (3.0)	549 (4.2)
North America	11 (0.9)	35 (1.3)	12 (4.4)	28 (0.6)	6 (0.5)	4 (1.2)	96 (0.7)
Missing	260 (20.6)	295 (11.3)	36 (1.2)	33 (0.7)	24 (2.2)	97 (29.5)	750 (5.8)
**Mode of HIV transmission**							
Heterosexual	68 (5.4)	1072 (41.2)	1758 (57.1)	2243 (50.5)	599 (45.4)	74 (22.5)	5814 (44.6)
Men who have sex with men/bisexual	727 (57.5)	1015 (34.0)	763 (24.8)	1369 (30.9)	443 (33.6)	47 (12.3)	4364 (33.5)
Intravenous drug use	340 (26.9)	268 (10.3)	240 (7.8)	201 (4.5)	35 (2.6)	9 (2.7)	1093 (8.4)
Mother to child	3 (0.2)	42 (1.6)	86 (2.8)	114 (2.6)	19 (1.4)	13 (3.9)	277 (2.1)
Blood products	104 (8.2)	49 (1.6)	37 (1.2)	34 (0.8)	8 (0.6)	2 (0.6)	234 (1.8)
Unknown/other	9 (0.7)	86 (3.3)	165 (5.3)	396 (8.9)	155 (11.7)	35 (10.6)	846 (6.5)
Missing	13 (1.0)	70 (2.7)	27 (0.9)	81 (1.8)	61 (4.6)	149 (45.3	401 (3.1)
**Level of immunosuppression**							
Nadir CD4 cell count, median (IQR)	90 (20-200)	150 (42-258)	204 (110-300)	278 (145-420)	300 (130-480)	385 (205-555)	218 (90-359)
Missing nadir CD4 cell count, n (%)	149 (11.8)	198 (7.6)	21 (0.7)	28 (0.6)	8 (0.6)	66 (20)	475 (3.6)

Data are numbers and percentages (%) unless otherwise stated. Percentages do not always add up to hundred due to rounding. Birth regions according to UNAIDS definitions.
