# Supplementary material for: Cohort profile: InfCareHIV, a prospective registry-based cohort study of people with diagnosed HIV in Sweden
Source: BMJ Open. 2023 Mar 17;13(3):e069688. doi: 10.1136/bmjopen-2022-069688 (PMC10030896; doi:10.1136/bmjopen-2022-069688)
Supplement: Supplementary data [file bmjopen-2022-069688supp002.pdf]

Supplementary Table 2. Summary of variables included in InfCareHIV

**Demographics**	
general	age, sex at birth, gender identity, ethnicity, country of birth
HIV-specific	mode of HIV-transmission, first ever positive HIV-serology, first positive HIV-serology in Sweden, suspected country of HIV transmission, last negative HIV-test, confirmed primary HIV infection
**Laboratory results**	
HIV-specific	HIV-1, HIV-2
	CD4+ count (absolute, %), CD8+ count (absolute, %), CD4/CD8 ratio, nadir CD4 count
	HIV-RNA copies /mL (plasma, CSF, sperm)
	genotypes, HIV sequences, elite-controllers
	HLA-B5701-allele
Other laboratory results	hepatitis A, B, C and D serologies, HBV-DNA, HCV-RNA
	syphilis serology
	SARS-CoV-2 (PCR)
Physical measurements	Weight, blood pressure etc.
**Treatment**	
Antiretroviral therapy	Start and stop dates, regimen (including dose and mode of administration), reason for change
Selected other medications	PCP-prophylaxis, Hepatitis C treatment etc.
**AIDS-diagnoses**	date and type of AIDS-diagnosis
**Co-morbidities**	diabetes type 1 and 2, hypertension, kidney failure, ischemic heart disease etc.
**Pregnancies**	dates of pregnancy, date of delivery etc.
**Death**	date and reason of death
**Health Questionnaire** *PROMs & PREMs*	Satisfaction with physical, psychological, and sexual health. Adherence to ART. Side effects. Smoking-habits. Satisfaction and involvement of care.

AIDS: acquired immunodeficiency syndrome, ART: antiretroviral therapy, CSF: cerebrospinal fluid, HBV: hepatis B virus, HCV: hepatitis C virus, HIV: human immunodeficiency virus, PCP: pneumocystis carinii prophylaxis, PROM: patient-reported outcome measures, PREM: patient-reported experience measures
