# Supplementary material for: Cohort profile: InfCareHIV, a prospective registry-based cohort study of people with diagnosed HIV in Sweden
Source: BMJ Open. 2023 Mar 17;13(3):e069688. doi: 10.1136/bmjopen-2022-069688 (PMC10030896; doi:10.1136/bmjopen-2022-069688)
Supplement: Supplementary data [file bmjopen-2022-069688supp003.pdf]

Health survey Quality registry InfCareHIV

Personal number:
Birth year:
Name:

Please tick the appropriate box for each statement

1. How satisfied are you with your physical health?

Very unsatisfied	Unsatisfied	Rather unsatisfied	Rather satisfied	Satisfied	Very satisfied
☐	☐	☐	☐	☐	☐

2. How satisfied are you with your psychological wellbeing?

Very unsatisfied	Unsatisfied	Rather unsatisfied	Rather satisfied	Satisfied	Very satisfied
☐	☐	☐	☐	☐	☐

3. How satisfied are you with your sexual life (regardless if you have sex with a partner or on your own)?

Very unsatisfied	Unsatisfied	Rather unsatisfied	Rather satisfied	Satisfied	Very satisfied
☐	☐	☐	☐	☐	☐

4a. Are you currently taking HIV medication?

Yes ☐à Go to question 4b. No ☐ à Go to question 5.

4b. Do you experience any side effects?

Yes ☐à Go to question 4c. No ☐à Go to question 4d.

4c. To what extent are you troubled by medical side effects?

Very troubled	Troubled	Rather troubled	Not very troubled	Not at all troubled
☐	☐	☐	☐	☐

4d. How many doses have you missed the last week?

0	1-2	3 or mote doses
☐	☐	☐

5. Do you smoke?

Never	Quitted	Yes
☐	☐	☐

6. Do you feel involved in the planning and realization of your HIV care and treatment?

Never	Seldom	Sometimes	Always
☐	☐	☐	☐

7. How satisfied are you with the quality of care provided at your HIV clinic?

Very unsatisfied	Unsatisfied	Rather unsatisfied	Rather satisfied	Satisfied	Very satisfied
☐	☐	☐	☐	☐	☐

Thank you for your participation!
