# Supplementary material for: Cohort profile: InfCareHIV, a prospective registry-based cohort study of people with diagnosed HIV in Sweden
Source: BMJ Open. 2023 Mar 17;13(3):e069688. doi: 10.1136/bmjopen-2022-069688 (PMC10030896; doi:10.1136/bmjopen-2022-069688)
Supplement: Author's manuscript [file bmjopen-2022-069688.draft_revisions.pdf]

BMJ Open

BMJ Open is committed to open peer review. As part of this commitment we make the peer review history of every article we publish publicly available.

When an article is published we post the peer reviewers' comments and the authors' responses online. We also post the versions of the paper that were used during peer review. These are the versions that the peer review comments apply to.

The versions of the paper that follow are the versions that were submitted during the peer review process. They are not the versions of record or the final published versions. They should not be cited or distributed as the published version of this manuscript.

BMJ Open is an open access journal and the full, final, typeset and author-corrected version of record of the manuscript is available on our site with no access controls, subscription charges or pay-per-view fees (http://bmjopen.bmj.com).

If you have any questions on BMJ Open's open peer review process please email info.bmjopen@bmj.com

BMJ Open

Cohort profile: InfCareHIV, a prospective registry-based cohort study of people with diagnosed HIV in Sweden

Journal:	*BMJ Open*
Manuscript ID	bmjopen-2022-069688
Article Type:	Cohort profile
Date Submitted by the Author:	29-Oct-2022
Complete List of Authors:	Carlander, Christina; Karolinska University Hospital, Department of Infectious Diseases; Karolinska Institute, Department of Medicine Huddinge<br>Brännström, Johanna; Karolinska Institutet, Department of Medicine Huddinge; Södersjukhuset, Department of Infectious Diseases/Venhälsan<br>Mansson, Fredrik; Infectious Diseases Research Unit, Department of Clinical Sciences, Lund University<br>Elvstam, Olof; Lund University, Department of Translational Medicine; Växjö Central Hospital, Department of Infectious Diseases<br>Albinsson, Pernilla; Karolinska University Hospital<br>Blom, Simon; HIV Sweden<br>Mattsson, Lena; Karolinska University Hospital<br>Hovmöller, Sanne; Sunderby Hospital, Department of Infectious Diseases<br>Norrgren, Hans; Lund University Faculty of Science<br>Mellgren, Aasa; Sahlgrenska University Hospital, Department of Infectious Diseases<br>Svedhem, Veronica; Karolinska University Hospital, Department of Infectious Diseases; Karolinska Institutet, Department of Medicine Huddinge<br>Gisslén, Magnus; University of Gothenburg Sahlgrenska Academy, Department of Infectious Diseases, Institute of Biomedicine; Sahlgrenska University Hospital, Department of Infectious Diseases, Region Västra Götaland<br>Sonnerborg, Anders; Division of Infectious Diseases, Department of Medicine Huddinge, Karolinska Institutet and Division of Clinical Microbiology, Department of Laboratory Medicine, Karolinska Institute
Keywords:	HIV & AIDS < INFECTIOUS DISEASES, Epidemiology < INFECTIOUS DISEASES, PREVENTIVE MEDICINE

SCHOLARONE™
Manuscripts

I, the Submitting Author has the right to grant and does grant on behalf of all authors of the Work (as defined in the below author licence), an exclusive licence and/or a non-exclusive licence for contributions from authors who are: i) UK Crown employees; ii) where BMJ has agreed a CC-BY licence shall apply, and/or iii) in accordance with the terms applicable for US Federal Government officers or employees acting as part of their official duties; on a worldwide, perpetual, irrevocable, royalty-free basis to BMJ Publishing Group Ltd ("BMJ") its licensees and where the relevant Journal is co-owned by BMJ to the co-owners of the Journal, to publish the Work in this journal and any other BMJ products and to exploit all rights, as set out in our licence.

The Submitting Author accepts and understands that any supply made under these terms is made by BMJ to the Submitting Author unless you are acting as an employee on behalf of your employer or a postgraduate student of an affiliated institution which is paying any applicable article publishing charge ("APC") for Open Access articles. Where the Submitting Author wishes to make the Work available on an Open Access basis (and intends to pay the relevant APC), the terms of reuse of such Open Access shall be governed by a Creative Commons licence – details of these licences and which Creative Commons licence will apply to this Work are set out in our licence referred to above.

Other than as permitted in any relevant BMJ Author's Self Archiving Policies, I confirm this Work has not been accepted for publication elsewhere, is not being considered for publication elsewhere and does not duplicate material already published. I confirm all authors consent to publication of this Work and authorise the granting of this licence.

For peer review only

BMJ Open Cohort profile

Title: Cohort profile: InfCareHIV, a prospective registry-based cohort study of people with diagnosed HIV in Sweden

Authors Christina Carlander[1,2], Johanna Brännström[2,3], Fredrik Månsson[4], Olof Elvstam[4,5], Pernilla Albinsson[1], Simon Blom[6], Lena Mattsson[1], Sanne Hovmöller[7], Hans Norrgren[8], Åsa Mellgren[9,10], Veronica Svedhem[1,2], Magnus Gisslén[9,10], Anders Sönnerborg[1,2]

Author affiliations

[1]Department of Infectious Diseases, Karolinska University Hospital, Stockholm, Sweden
[2]Division of Infectious Diseases, Department of Medicine Huddinge, Karolinska Institutet, Stockholm, Sweden
[3]Department of Infectious Diseases/Venhälsan, Södersjukhuset, Stockholm, Sweden
[4]Department of Translational Medicine, Lund University, Malmö, Sweden
[5]Department of Infectious Diseases, Växjö Central Hospital, Växjö, Sweden
[6]Ombudsman, HIV-Sweden, Stockholm, Sweden
[7]Department of Infectious Diseases, Sunderby Hospital, Luleå
[8]Department of Clinical Sciences Lund, Lund University, Lund, Sweden
[9]Department of Infectious Diseases, Institute of Biomedicine, Sahlgrenska Academy, University of Gothenburg
[10]Department of Infectious Diseases, Region Västra Götaland, Sahlgrenska University Hospital, Gothenburg, Sweden

For peer review only

Abstract

Purpose The Swedish InfCareHIV cohort was established in 2003 to ensure equal and effective care of people living with HIV (PLHIV) and enable long-term follow-up. InfCareHIV functions equally as a decision support system as a quality registry, ensuring up-to-date data reported in real time.

Participants InfCareHIV includes data on >99% of all people with diagnosed HIV in Sweden and up to now 13,029 have been included in the cohort. InfCareHIV includes data on HIV-related biomarkers and antiretroviral therapies (ART) and also on demographics, patient-reported outcome measures and patient-reported experience measures.

Findings to date: Sweden was in 2015 the first country to reach the UNAIDS/WHO's 90-90-90 goals. Late diagnosis of HIV infection was identified as a key problem in the Swedish HIV-epidemic, and low-level HIV viremia while on ART associated to all-cause mortality. Increased HIV RNA load in the cerebrospinal fluid despite suppression of the plasma viral load was found in 5% of PLHIV, a phenomenon referred to as "CSF viral escape".

Dolutegravir-based treatment in PLHIV with pre-existing NRTI-mutations was non-inferior to protease inhibitor-based regimens. An increase of transmitted drug resistance was observed in the InfCareHIV cohort. Lower efficacy for protease inhibitors was not due to lower adherence to treatment. Incidence of type 2 diabetes and insulin resistance was high in the aging HIV population. Despite ART, the risk of infection-related cancer as well as lung cancer was increased in PLHIV compared to HIV-negative. PLHIV were less likely successfully treated for cervical precancer and more likely to have human papillomavirus types not included in current HPV vaccines. Self-reported sexual satisfaction in PLHIV is improving and was higher in women than men.

Future plans InfCareHIV provides a unique base to study and further improve long-term treatment outcomes, comorbidity management and health-related quality of life in people with HIV in Sweden.

For peer review only

For peer review only - http://bmjopen.bmj.com/site/about/guidelines.xhtml

Introduction

With access to antiretroviral therapy (ART) the overall life expectancy of people living with HIV (PLHIV) is close to that of HIV-negative people[1]. However, studies from the Swedish InfCareHIV cohort show that after a 15-year follow-up period, successfully treated PLHIV in Sweden were three times more likely to die when compared with HIV negative controls[2]. The InfCareHIV cohort is heterogenous, including a large proportion of migrants from 108 countries on all continents, diverse modes of HIV transmission and socioeconomic backgrounds and is gender balanced. The *pol* sequence of the virus is characterized at diagnosis regarding drug resistance mutations and subtype. All known HIV-1 subtypes, many recombinants as well as unique recombinant forms and HIV-2 are represented. Pre-treatment HIV drug resistance is a critical aspect that requires global collaborative studies and to minimize its effect, retention in care and optimal adherence to treatment is essential[3]. The cohort is aging and faced with increasing risk of age-related comorbidities. Meanwhile, chronic HIV-related inflammation, legacy of pre-treatment immunodeficiency and injury, and past and present ART may also add to the development of comorbidity[4,5]. Meanwhile, half of PLHIV are still diagnosed late with well-known consequences for prognosis and transmission[6]. Despite that almost all PLHIV reaches treatment goals of undetectable plasma viral levels, health-related quality of life (HRQoL) is poorer compared with HIV-negative people[7,8]. This article describes the prospective Swedish HIV cohort, InfCareHIV, including all diagnosed people living with HIV in Sweden, with data dating back to the earliest cases in the 1980ies.

Cohort description

Study population

The InfCareHIV cohort was established in 2003 in the two largest cities in Sweden, Stockholm, and Gothenburg. These two cities care for about half of all PLHIV in Sweden. The other Swedish clinics joined thereafter and since 2008 the cohort has had complete national coverage, including all 29 clinical HIV centres in Sweden. To date (August 2022), 13 029 PLHIV have been included of whom 8,436 are currently in active care (Table 1). Of those in active care 39% are women and 1.2% are children below 18 years of age. A majority was born outside Sweden (67%), most commonly in sub-Saharan Africa (36%). About half (51%) have stated heterosexual mode of transmission, about a third are men who have sex with men or bisexual men (31%). Only 4% have stated intravenous drug use as mode of transmission while 3% were mother-to-child transmissions, 1.4% through blood products and for 9.6%

For peer review only - http://bmjopen.bmj.com/site/about/guidelines.xhtml

transmission mode is unknown or information is missing (Table 1). Almost all are on ART (98%), and of them almost everybody (95%) reaches the treatment goal of HIV-RNA<50 copies/mL (98% HIV-RNA <200 copies/mL) in snapshot analysis after at least six months of treatment[9]. Of the 4,483 people no longer on follow-up in InfCareHIV, 56% are deceased, 34% have emigrated and 3% are lost-to follow-up. InCareHIV also includes undocumented migrants who often end-up as lost-to follow-up (Table 1). Retrospective data from before the cohort was established (1983-2007) have been backlogged including PLHIV deceased before 2008.

Coverage and validation of the study population

The national coverage is nearly 100% as all 29 clinical centres that attend PLHIV are included in InfCareHIV, and more than 99% of all diagnosed PLHIV who are living in Sweden are included. This number has been repeatedly validated, most recently in 2019, against the number of HIV-diagnoses reported to the Public Health Agency of Sweden, a report that is mandatory by Swedish law. The validations found that data regarding HIV diagnoses were even more accurate in InfCareHIV than in the Public Health Agencies records.

Cohort variables

Variables collected and manually registered by a health professional at enrolment include sex at birth, gender identity (added 2021), country of birth, mode of HIV transmission, date of any last negative HIV-test and first positive HIV-test (in Sweden and if relevant abroad), any AIDS diagnoses, confirmed primary HIV infection and suspected country of HIV transmission (Supplementary Table 1). Data collected/updated at each follow-up visit include ART start and stop dates, ART regimen (including doses and mode of administration) and the reason for any change of drug regimens, prophylaxis of opportunistic infections and selected co-medications. Serological and virological data on co-infection with hepatitis C and B virus, weight, date and type of AIDS-defining events, date and cause of death are also included. HIV-RNA (plasma and cerebrospinal fluid [CSF]) and HIV drug resistance (including viral sequences) results as well as CD4+ and CD8+ T-cell counts and CD4/CD8 ratios are either automatically or manually included depending on the HIV centre. HIV subtype and HLA B-5701-allele are registered (Supplementary Table 1). Pregnancies and following deliveries are also registered.

Validation of cohort variables

For peer review only - http://bmjopen.bmj.com/site/about/guidelines.xhtml

Validation of data is performed regularly on national level using a Data Quality Index, with the possibility to validate also on clinic and individual level. Data Quality Index for InfCareHIV is currently at 4.9 on a 0-5 scale. Data are also validated through the Distributed Data Management tool which enables data extraction for data sharing and automatically conducts quality assurance checks that signal any incorrect values that may then be manually corrected. This tool is derived and modified from a collaboration with EuroCoord, the European Network of HIV/AIDS Cohort studies to coordinate at European and International Level Clinical Research on HIV/AIDS[10].

Ethics

At time of entering HIV care, PLHIV are informed about the registry, after which they can either give verbal consent or opt out. Participants always have the right to exit InfCareHIV, although this has been very uncommon. Participants can request an extract on their data from the registry, free of charge, in accordance with the European General Data Protection Regulation (GDPR 2016/679) and the Swedish Data Protection Act (2018:218).

Patient and public involvement

A representative from a Swedish organization for PLHIV is co-opted to the InfCareHIV steering committee. A webpage (www.infcarehiv.se) contains information for both PLHIV, professionals involved in HIV care, researchers, and the public. Statistics and treatment results of the individual clinical centres are published openly in an annual report and on the web page, more on that below.

Decision support system

The mainstay of InfCareHIV is the clinical decision support system (Figure 1). Data on HIV-RNA levels (plasma and CSF), CD4 cell counts (absolute and percentage), drug resistance, and previous and current ART are presented through a graphical system. In addition, the legend below the graph presents data regarding date of HIV diagnosis, date of first ART and names of the HIV-team consisting of doctor, nurse, and counsellor. Periods of pregnancy are also shown in the graph. All other information is easily accessible in the system. Also, since all viral (pol) sequences are stored in the database, reanalysis can easily be performed when new drugs or new information about resistance appear. Thus, data generated in clinical care are automatically "reused" for research and development. The graph gives the clinician a relevant visual summary that is easier to grasp than the medical records, especially in

For peer review only - http://bmjopen.bmj.com/site/about/guidelines.xhtml

treatment experienced PLHIV with a long history of different ART. Meanwhile the graph is also a pedagogical instrument during patient consultation, facilitating patient-centered care, making it easy to illustrate for example the consequences of good or poor treatment adherence. HRQoL is also illustrated in the graph. Finally, the graph is used when transferring PLHIV between clinic centres, for expert-consultancy and at multi-disciplinary team conferences.

Quality Registry

An important feature of InfCareHIV is to function as a National Quality Registry. The aim of a quality registry is to develop and ensure quality of care systematically and continuously. This allows for comparisons on HIV care at a national, regional, and clinical centre level and has led to increased equality of care on national level with diminishing differences in treatment results in Sweden's HIV centres. We believe it is most likely that this contributed to that Sweden in 2015 was the first country to reach the United Nations Programme on HIV/AIDS/World Health Organization´s 90-90-90 goal with 90% diagnosed, 90% of them on ART and 90% of them with viral suppression[9]. In concordance with WHO/UNAIDS updated goal, we are now measuring the 95-95-95 goal and have also added 95 goals for HRQoL.

Health-related quality of life

Systematical quantification of patient-reported outcomes (PROs) will assist the improvement of medical care and HRQoL of PLHIV. In 2011, a 9-item self-reported Health Questionnaire (HQ) was integrated to the registry to be answered annually by PLHIV either via a website or using a computerized or paper version at the outpatient clinic (Supplementary Table 2) [11]. The questionnaire was developed together with representatives from PLHIV patient organizations. The HQ assesses patient-reported outcome measures (PROMs) regarding physical, psychological, and sexual health, self-reported ART adherence, experience of side effects and patient-reported experience measures (PREMs) regarding involvement and satisfaction with care. A question on smoking habits was added in 2017. The questionnaire is electronically available in Swedish and English and in paper version in the eight most common languages in the InfCareHIV cohort. Illiterate patients are offered help by a nurse/interpreter. The results are presented in the Decision support system (Figure 1), where red symbolises low ratings that need attention, yellow medium ratings, and green contentment. The HQ is validated by content validity and test-retest reliability and has also been evaluated as a tool for longitudinal follow up of trends in PROs[11,12].Our fourth 95 goal is that 95% of all PLHIV should perform

For peer review only - http://bmjopen.bmj.com/site/about/guidelines.xhtml

the health questionnaire and that 95% of PLHIV should have an ART regimen without experience of side-effects.

HIV-related stigma and discrimination are major obstacles for reaching good HRQoL. In close relation to InfCareHIV we developed a 12-item HIV stigma scale, a short version of the commonly used 40-item HIV Stigma Scale by Berger et al[13]. It is a valid and reliable instrument for the measurement of enacted, anticipated, and internalized stigma and crucial for mapping trends in the prevalence of HIV-related stigma and tracking the effectiveness of stigma-reducing interventions[13,14].

Biobank

In the major clinical centres blood plasma/serum/liquor samples are collected, and in selected cases also peripheral blood mononuclear cells, HIV isolates, CSF and CSF cells in biobanks. These biobanks are separate from the cohort but linking between the cohort data and the biobanks can be performed (after ethical permission) for translational studies.

Selected findings from the past ten years to date

In a national prospective study, using InfCareHIV linked to patient study forms, Brännström et al. found late diagnosis of HIV infection to be a key problem in the Swedish HIV-1 epidemic, where more than half of the patients were diagnosed late, but the majority could have been diagnosed earlier with a more efficient health care system[6,15,16]. Most of the patients experienced barriers to HIV testing, but less so if the HIV-test was offered through screening programs or by a health care professional rather than having to be self-initiated[17,18]. Elvstam et al. reported an association between low-level HIV viremia while on ART and all-cause mortality[19], and has further explored the effects of detectable viremia during ART by linking InfCareHIV to National Health registries and analyzing stored biobank samples[20-24]. Malmström et al. studied risk of cancer by HIV status in Sweden for three decades, finding that PLHIV have a remaining increased risk of infection-related cancer despite ART, while lung cancer was the only non-infection-related cancer increased in PLHIV[25].

By linking InfCareHIV to the National Cervical Cancer Screening registry, Carlander et al. showed that PLHIV are less likely to have successful treatment of cervical precancer and more likely to have human papillomavirus (HPV) types not included in current HPV vaccines[26-29]. Brännström et al. showed that the incidence of type 2 diabetes and insulin resistance is high in the aging HIV population, where comorbidities are common and

For peer review only - http://bmjopen.bmj.com/site/about/guidelines.xhtml

associated whereas there was no association found for HIV related factors[30]. Möller et al. showed that well-treated PLHIV are not at higher odds of severe COVID-19 compared with HIV-negative people after controlling for age and comorbidity[31].

Sörstedt et al. studied the effect of dolutegravir-based treatment in PLHIV with pre-existing NRTI-mutations and found a non-inferior effect compared to protease inhibitor based regimens[32] and has also explored viral blips during ART[33]. Gisslén et al. have used InfCareHIV in a substantial number of studies exploring CSF viral load and other biomarkers in different settings of untreated and treated HIV[34-52]. Viral load is normally 1 log lower in CSF than in plasma in untreated HIV[53], but CSF exceeds plasma HIV RNA in approximately 15% of patients, with considerable variations between different disease stages[34]. CNS infection is generally well controlled by systemic suppressive ART[54], although in approximately 5% the HIV RNA load was increased in the CSF despite suppression of the plasma viral load, a phenomenon referred to as "CSF viral escape"[39]. This phenomenon is most often transient, not associated with any symptoms and comparable to plasma viral blips[55] which occur in about the same frequency[33]. Several studies have indicated that that a stable, permanent infection of cells in the CNS is established later than in systemic viral reservoirs[38], which has implications when exploring HIV eradication strategies[56]. Sönnerborg et al have extensively studied, since the 1990-thies, long-term nonprogressors and elite controllers (EC), selected from the InfCareHIV cohort. Specific immunological[57] and metabolic features have been described[58]. Also a naturally occurring dipeptide was found to be enhanced among EC and to possess antiretroviral properties, acting as both an entry inhibitor and an RT-inhibitor[59]. Sönnerborg et al have also studied HIV drug resistance, both transmitted and acquired, and molecular epidemiology. Among several key findings, an increase of transmitted drug resistance in the InfCareHIV cohort[3] was reported. A pronounced HIV-1 subtype heterogeneity, including all known subtypes, many circulating recombinant forms and unique recombinant forms was described in the InfCareHIV cohort[60].

Svedhem et al. validated the Health Questionnaire and demonstrated that self-reported adherence was correlated to viral suppression and described determinants of optimal ART-adherence in the cohort[11]. Svedhem et al. have shown that the assessment of PROMs is an important tool to ensure the long-term adherence to treatment, improvement in quality of life and evaluate side-effects on HIV treatment[11,61]. PROMs have also successfully been used in virological molecular modeling showing that lower affinity for protease inhibitors to HIV-1C

For peer review only - http://bmjopen.bmj.com/site/about/guidelines.xhtml

protease do not depend on lower adherence to treatment among people infected with subtype C[12].

Tyberg et al. compared plasma drug levels of ART, potential drug-drug interactions and side-effects in PWH aged ≥ 65 years of age, with controls ≤ 49 years of age, and found differences in drug concentrations and reported side effects between groups[62]. Mellgren et al demonstrated that the experience of side-effects of ART declined significantly in the cohort during 2011-2017 and that experiences of side effects were diverse and associated with both self-reported physical and psychological health[61]. Two studies on self-reported sexuality by Mellgren et al. found that self-reported sexual satisfaction in PLHIV improved annually and that women were more satisfied with their sexual life compared with men[63,64]. In women living with HIV, satisfaction with sexual life was associated with self-reported psychological health and experiences of side-effects. Carlander et al. showed that over the past twenty years access to employment has increased in PLHIV although remaining lower compared to HIV-negative, even after controlling for migrant and socioeconomic status[65].

International collaborations

InfCareHIV has collaborated or collaborates with several international HIV cohorts such as EuroCoord, EuroSIDA, PENTA, COHERE, CASCADE, NEAT, EuResist, EuCare, RESPOND, UCSF CSF Cohort, and CARE.

Future plans

InfCareHIV provides a unique base to continue study long-term treatment, comorbidities and HRQoL of people with HIV in Sweden. Half of all PLHIV is still diagnosed late and studies that can help improve HIV-testing guidelines and HIV awareness are essential. Attempts are made to develop more precise bioinformatics tools for assessment of time of infection and the undiagnosed population. Pre-treatment HIV drug resistance is a critical aspect that requires global collaboration and long-term follow-up. Despite most PLHIV reaching the treatment goal of undetectable viral level, patient reported health is still poorer than for HIV-negative people and studies on how to improve HRQoL in PLHIV and minimize stigma should be prioritized. With our aging cohort, studies on comorbidity becomes more important and specially to assess what comorbidities are associated with normal aging and what comorbidities may be associated with ART or HIV-induced chronic inflammation. Multiomics using samples and clinical information is presently evaluated to identify biomarkers for prediction of comorbidities, to characterize the evolution of central nervous

For peer review only - http://bmjopen.bmj.com/site/about/guidelines.xhtml

system (CNS) inflammation and injury[43] and for future selection of patients in HIV cure attempts.

Strengths and limitations

The main strength of the InfCareHIV cohort is that it includes all diagnosed PLHIV in Sweden. The accuracy, completeness, consistency and validity is very high and consequently InfCareHIV has the highest certification level among Swedish Health Quality registries according to the Swedish Association of Local Authorities and Regions[66]. The personal identity number given to all Swedish residents enables linkage of data to any Swedish population or health registry after appropriate application, providing opportunities to answer a wide range of research questions. Also, the automatic transfer of key laboratory data, including viral sequences, and the possibility of linking cohort data and biobanks allow easy reuse of information obtained in clinical care for research purposes. Some variables in InfCareHIV (e.g., comorbidity, weight and self-reported smoking status that has been added in later years) lacks in coverage which currently limits their use in research but linking to other health registries for the collection of this data is then a possibility. The aim is for all PLHIV to be invited to answer the self-reported health questionnaire annually to facilitate patient centered care and improve HRQOL, although currently we reach only about 34%, and it is a prioritized matter to improve this number.

Collaboration We encourage collaborations with HIV cohorts from other countries. Data can be made available upon reasonable request and after ethical approval.

Further details

Author affiliations

[1]Department of Infectious Diseases, Karolinska University Hospital, Stockholm, Sweden
[2]Division of Infectious Diseases, Department of Medicine Huddinge, Karolinska Institutet, Stockholm, Sweden
[3]Department of Infectious Diseases/Venhälsan, Södersjukhuset, Stockholm, Sweden
[4]Department of Translational Medicine, Lund University, Malmö, Sweden
[5]Department of Infectious Diseases, Växjö Central Hospital, Växjö, Sweden
[6]Ombudsman, HIV-Sweden, Stockholm, Sweden
[7]Department of Infectious Diseases, Sunderby Hospital, Luleå
[8]Department of Clinical Sciences Lund, Lund University, Lund, Sweden
[9]Department of Infectious Diseases, Institute of Biomedicine, Sahlgrenska Academy, University of Gothenburg
[10]Department of Infectious Diseases, Region Västra Götaland, Sahlgrenska University Hospital, Gothenburg, Sweden

For peer review only

Acknowledgments First of all, the authors would like to thank all people living with HIV in Sweden enrolled in the cohort. We also thank all clinical centres that work daily with the inclusion and update of data in InfCareHIV.

Contributors CC drafted the manuscript. All authors critically revised the manuscript.

Funding No funding to report for this paper.

Competing interests CC has received lecture, moderator, and advisory board fees from GSK/ViiV, Gilead Sciences and MSD and an unrestricted Nordic Fellowship Grant from Gilead Sciences Nordic. JB has received lecture and advisory board fees from GSK/ViiV and Gilead. FM has received lecture and advisory board fees from GSK/ViiV, Astra Zeneca and Gilead Sciences. OE has received a grant to his institution from Pfizer and honoraria as speaker from Gilead Sciences. HN has received advisory board fees from Gilead and Abbvie. ÅM has received lecture and advisory board fees from GSK/ViiV, Gilead Sciences and Pfizer. MG has received research grants from Gilead Sciences and Janssen-Cilag and honoraria as speaker, DSMB committee member and/or scientific advisor from Amgen, AstraZeneca Biogen, Bristol-Myers Squibb, Gilead Sciences, GlaxoSmithKline/ViiV, Janssen-Cilag, MSD, Novocure, Novo Nordic, Pfizer and Sanofi. AS has received research grants from Gilead Sciences and honoraria as speaker, DSMB committee member and/or scientific advisor from AstraZeneca, Bristol-Myers Squibb, Gilead Sciences, GlaxoSmithKline/ViiV, and MSD. All other authors declare no competing interests

Patient consent for publication Not applicable.

Ethics approval This cohort study has been approved by the Regional Ethical committees in Sweden (Dnr 532-11, Dnr 2018/11-31/2, Dnr 2022-02897-02).

Provenance and peer review Not commissioned, externally peer reviewed.

Data availability statement Data can be made available upon reasonable request and after ethical approval.

References

1. Survival of HIV-positive patients starting antiretroviral therapy between 1996 and 2013: a collaborative analysis of cohort studies. *The lancet HIV*. Aug 2017;4(8):e349-e356. doi:10.1016/s2352-3018(17)30066-8

2. de Coninck Z, Hussain-Alkhateeb L, Bratt G, et al. Non-AIDS Mortality Is Higher Among Successfully Treated People Living with HIV Compared with Matched HIV-Negative Control Persons: A 15-Year Follow-Up Cohort Study in Sweden. *AIDS Patient Care STDS*. 08 2018;32(8):297-305. doi:10.1089/apc.2018.0015

For peer review only

3. Andersson E, Ambikan A, Brännström J, et al. High-throughput sequencing reveals a high prevalence of pretreatment HIV-1 drug resistance in Sweden. *AIDS (London, England)*. Feb 2 2021;35(2):227-234. doi:10.1097/qad.0000000000002740

4. Schnittman SR, Deitchman AN, Beck-Engeser G, et al. Abnormal Levels of Some Biomarkers of Immune Activation Despite Very Early Treatment of Human Immunodeficiency Virus. *The Journal of infectious diseases*. May 20 2021;223(9):1621-1630. doi:10.1093/infdis/jiaa580

5. Gabuzda D, Jamieson BD, Collman RG, et al. Pathogenesis of Aging and Age-related Comorbidities in People with HIV: Highlights from the HIV ACTION Workshop. *Pathogens & immunity*. 2020;5(1):143-174. doi:10.20411/pai.v5i1.365

6. Brannstrom J, Svedhem Johansson V, Marrone G, et al. Deficiencies in the health care system contribute to a high rate of late HIV diagnosis in Sweden. *HIV medicine*. Jun 2016;17(6):425-35. doi:10.1111/hiv.12321

7. Lazarus JV, Safreed-Harmon K, Barton SE, et al. Beyond viral suppression of HIV - the new quality of life frontier. *BMC Med*. 06 22 2016;14(1):94. doi:10.1186/s12916-016-0640-4

8. Miners A, Phillips A, Kreif N, et al. Health-related quality-of-life of people with HIV in the era of combination antiretroviral treatment: a cross-sectional comparison with the general population. *Lancet HIV*. Oct 2014;1(1):e32-40. doi:10.1016/S2352-3018(14)70018-9

9. Gisslén M, Svedhem V, Lindborg L, et al. Sweden, the first country to achieve the Joint United Nations Programme on HIV/AIDS (UNAIDS)/World Health Organization (WHO) 90-90-90 continuum of HIV care targets. *HIV medicine*. Apr 2017;18(4):305-307. doi:10.1111/hiv.12431

10. Commission E. European network of HIV/AIDS cohort studies to coordinate at European and International Level Clinical Research on HIV/AIDS. 24th August, 2022. Accessed 24th August, 2022. https://cordis.europa.eu/project/id/260694

11. Marrone G, Mellgren Å, Eriksson LE, et al. High Concordance between Self-Reported Adherence, Treatment Outcome and Satisfaction with Care Using a Nine-Item Health Questionnaire in InfCareHIV. *PloS one*. 2016;11(6):e0156916. doi:10.1371/journal.pone.0156916

12. Häggblom A, Svedhem V, Singh K, et al. Virological failure in patients with HIV-1 subtype C receiving antiretroviral therapy: an analysis of a prospective national cohort in Sweden. *Lancet HIV*. Apr 2016;3(4):e166-74. doi:10.1016/S2352-3018(16)00023-0

13. Reinius M, Wettergren L, Wiklander M, et al. Development of a 12-item short version of the HIV stigma scale. *Health Qual Life Outcomes*. May 30 2017;15(1):115. doi:10.1186/s12955-017-0691-z

14. Reinius M, Wiklander M, Wettergren L, et al. The Relationship Between Stigma and Health-Related Quality of Life in People Living with HIV Who Have Full Access to Antiretroviral Treatment: An Assessment of Earnshaw and Chaudoir's HIV Stigma Framework Using Empirical Data. *AIDS Behav*. Dec 2018;22(12):3795-3806. doi:10.1007/s10461-018-2041-5

15. Brännström J, Svedhem V, Marrone G, et al. Symptomatic Patients without Epidemiological Indicators of HIV Have a High Risk of Missed Diagnosis: A Multi-Centre Cross Sectional Study. *PLoS One*. 2016;11(9):e0162503. doi:10.1371/journal.pone.0162503

16. Brännström J, Sönnerborg A, Svedhem V, et al. A high rate of HIV-1 acquisition post immigration among migrants in Sweden determined by a CD4 T-cell decline trajectory model. *HIV Med*. 10 2017;18(9):677-684. doi:10.1111/hiv.12509

17. Wiklander M, Brännström J, Svedhem V, et al. Development and psychometric testing of a barriers to HIV testing scale among individuals with HIV infection in Sweden;

For peer review only - http://bmjopen.bmj.com/site/about/guidelines.xhtml

The Barriers to HIV testing scale-Karolinska version. *Health Qual Life Outcomes*. Nov 19 2015;13:185. doi:10.1186/s12955-015-0381-7
18. George-Svahn L, Eriksson LE, Wiklander M, et al. Barriers to HIV testing as reported by individuals newly diagnosed with HIV infection in Sweden. *AIDS Care*. 10 2021;33(10):1286-1295. doi:10.1080/09540121.2020.1844862
19. Elvstam O, Marrone G, Medstrand P, et al. All-Cause Mortality and Serious Non-AIDS Events in Adults With Low-level Human Immunodeficiency Virus Viremia During Combination Antiretroviral Therapy: Results From a Swedish Nationwide Observational Study. *Clin Infect Dis*. 06 15 2021;72(12):2079-2086. doi:10.1093/cid/ciaa413
20. Elvstam O, Medstrand P, Yilmaz A, et al. Virological failure and all-cause mortality in HIV-positive adults with low-level viremia during antiretroviral treatment. *PLoS One*. 2017;12(7):e0180761. doi:10.1371/journal.pone.0180761
21. Elvstam O, Medstrand P, Jansson M, et al. Is low-level HIV-1 viraemia associated with elevated levels of markers of immune activation, coagulation and cardiovascular disease? *HIV Med*. 10 2019;20(9):571-580. doi:10.1111/hiv.12756
22. Elvstam O, Marrone G, Medstrand P, et al. Associations Between Plasma Human Immunodeficiency Virus (HIV) Ribonucleic Acid Levels and Incidence of Invasive Cancer in People With HIV After Initiation of Combination Antiretroviral Therapy. *Open Forum Infect Dis*. Jun 2021;8(6):ofab131. doi:10.1093/ofid/ofab131
23. Brattgård H, Björkman P, Nowak P, et al. Factors associated with low-level viraemia in people with HIV starting antiretroviral therapy: A Swedish observational study. *PLoS One*. 2022;17(5):e0268540. doi:10.1371/journal.pone.0268540
24. Elvstam O, Marrone G, Engström G, et al. Associations between HIV viremia during antiretroviral therapy and cardiovascular disease. *AIDS*. Jun 23 2022;doi:10.1097/QAD.0000000000003240
25. Malmström S, Wagner P, Yilmaz A, et al. Failure to restore CD4+ cell count associated with infection-related and noninfection-related cancer. *AIDS (London, England)*. Mar 1 2022;36(3):447-457. doi:10.1097/qad.0000000000003117
26. Carlander C, Wagner P, van Beirs A, et al. Suppressive antiretroviral therapy associates with effective treatment of high-grade cervical intraepithelial neoplasia. *AIDS (London, England)*. Jul 17 2018;32(11):1475-1484. doi:10.1097/qad.0000000000001853
27. Carlander C, Wagner P, Svedhem V, et al. Impact of immunosuppression and region of birth on risk of cervical intraepithelial neoplasia among migrants living with HIV in Sweden. *Int J Cancer*. May 13 2016;doi:10.1002/ijc.30188
28. Carlander C, Lagheden C, Eklund C, et al. HPV Types in Cervical Precancer by HIV Status and Birth Region: A Population-Based Register Study. *Cancer epidemiology, biomarkers & prevention : a publication of the American Association for Cancer Research, cosponsored by the American Society of Preventive Oncology*. Dec 2020;29(12):2662-2668. doi:10.1158/1055-9965.epi-20-0969
29. Carlander C, Lagheden C, Eklund C, et al. Nonvaccine human papillomavirus genotype common in women with HIV failing cervical precancer treatment. *AIDS (London, England)*. Nov 15 2021;35(14):2367-2374. doi:10.1097/qad.0000000000003026
30. Bratt G, Brännström J, Missalidis C, et al. Development of type 2 diabetes and insulin resistance in people with HIV infection: Prevalence, incidence and associated factors. *PLoS One*. 2021;16(6):e0254079. doi:10.1371/journal.pone.0254079
31. Möller I, Gisslén M, Wagner P, et al. Clinical outcome in people with COVID-19 by HIV-status: a nationwide register study. presented at: CROI; 2022; Virtual. Session Poster-R01.
32. Sörstedt E, Carlander C, Flamholc L, et al. Effect of dolutegravir in combination with Nucleoside Reverse Transcriptase Inhibitors (NRTIs) on people living with HIV who

For peer review only

have pre-existing NRTI mutations. *Int J Antimicrob Agents*. May 2018;51(5):733-738. doi:10.1016/j.ijantimicag.2018.01.009
33. Sörstedt E, Nilsson S, Blaxhult A, et al. Viral blips during suppressive antiretroviral treatment are associated with high baseline HIV-1 RNA levels. *BMC Infect Dis*. 06 21 2016;16:305. doi:10.1186/s12879-016-1628-6
34. Ulfhammer G, Edén A, Antinori A, et al. Cerebrospinal Fluid viral load across the spectrum of untreated HIV-1 infection: a cross-sectional multi-center study. *Clin Infect Dis*. Nov 08 2021;doi:10.1093/cid/ciab943
35. Hagberg L, Price RW, Zetterberg H, et al. Herpes zoster in HIV-1 infection: The role of CSF pleocytosis in secondary CSF escape and discordance. *PLoS One*. 2020;15(7):e0236162. doi:10.1371/journal.pone.0236162
36. Joseph SB, Kincer LP, Bowman NM, et al. Human Immunodeficiency Virus Type 1 RNA Detected in the Central Nervous System (CNS) After Years of Suppressive Antiretroviral Therapy Can Originate from a Replicating CNS Reservoir or Clonally Expanded Cells. *Clin Infect Dis*. 09 27 2019;69(8):1345-1352. doi:10.1093/cid/ciy1066
37. Ulfhammer G, Edén A, Mellgren Å, et al. Persistent central nervous system immune activation following more than 10 years of effective HIV antiretroviral treatment. *AIDS*. 09 24 2018;32(15):2171-2178. doi:10.1097/QAD.0000000000001950
38. Burbelo PD, Price RW, Hagberg L, et al. Anti-Human Immunodeficiency Virus Antibodies in the Cerebrospinal Fluid: Evidence of Early Treatment Impact on Central Nervous System Reservoir? *J Infect Dis*. 03 13 2018;217(7):1024-1032. doi:10.1093/infdis/jix662
39. Edén A, Nilsson S, Hagberg L, et al. Asymptomatic Cerebrospinal Fluid HIV-1 Viral Blips and Viral Escape During Antiretroviral Therapy: A Longitudinal Study. *J Infect Dis*. Dec 15 2016;214(12):1822-1825. doi:10.1093/infdis/jiw454
40. Yilmaz A, Yiannoutsos CT, Fuchs D, et al. Cerebrospinal fluid neopterin decay characteristics after initiation of antiretroviral therapy. *J Neuroinflammation*. May 10 2013;10:62. doi:10.1186/1742-2094-10-62
41. Anesten B, Zetterberg H, Nilsson S, et al. Effect of antiretroviral treatment on blood-brain barrier integrity in HIV-1 infection. *BMC Neurol*. Dec 22 2021;21(1):494. doi:10.1186/s12883-021-02527-8
42. Yilmaz A, Mellgren Å, Fuchs D, et al. Switching from a regimen containing abacavir/lamivudine or emtricitabine/tenofovir disoproxil fumarate to emtricitabine/tenofovir alafenamide fumarate does not affect central nervous system HIV-1 infection. *Infect Dis (Lond)*. 2019 Nov - Dec 2019;51(11-12):838-846. doi:10.1080/23744235.2019.1670352
43. Gisslen M, Keating SM, Spudich S, et al. Compartmentalization of cerebrospinal fluid inflammation across the spectrum of untreated HIV-1 infection, central nervous system injury and viral suppression. *PLoS One*. 2021;16(5):e0250987. doi:10.1371/journal.pone.0250987
44. Hermansson L, Yilmaz A, Axelsson M, et al. Cerebrospinal fluid levels of glial marker YKL-40 strongly associated with axonal injury in HIV infection. *J Neuroinflammation*. Jan 24 2019;16(1):16. doi:10.1186/s12974-019-1404-9
45. Yilmaz A, Fuchs D, Price RW, et al. Cerebrospinal Fluid Concentrations of the Synaptic Marker Neurogranin in Neuro-HIV and Other Neurological Disorders. *Curr HIV/AIDS Rep*. 02 2019;16(1):76-81. doi:10.1007/s11904-019-00420-1
46. Gisslén M, Heslegrave A, Veleva E, et al. CSF concentrations of soluble TREM2 as a marker of microglial activation in HIV-1 infection. *Neurol Neuroimmunol Neuroinflamm*. 01 2019;6(1):e512. doi:10.1212/NXI.0000000000000512

For peer review only

47. Anesten B, Yilmaz A, Hagberg L, et al. Blood-brain barrier integrity, intrathecal immunoactivation, and neuronal injury in HIV. *Neurol Neuroimmunol Neuroinflamm*. Dec 2016;3(6):e300. doi:10.1212/NXI.0000000000000300
48. Ahlgren E, Hagberg L, Fuchs D, et al. Association between Plasma Homocysteine Levels and Neuronal Injury in HIV Infection. *PLoS One*. 2016;11(7):e0158973. doi:10.1371/journal.pone.0158973
49. Krut JJ, Price RW, Zetterberg H, et al. No support for premature central nervous system aging in HIV-1 when measured by cerebrospinal fluid phosphorylated tau (p-tau). *Virulence*. 07 04 2017;8(5):599-604. doi:10.1080/21505594.2016.1212155
50. Gisslén M, Price RW, Andreasson U, et al. Plasma Concentration of the Neurofilament Light Protein (NFL) is a Biomarker of CNS Injury in HIV Infection: A Cross-Sectional Study. *EBioMedicine*. Jan 2016;3:135-140. doi:10.1016/j.ebiom.2015.11.036
51. Karlsson U, Repits J, Antonsson L, et al. Reduced Baseline Sensitivity to Maraviroc Inhibition Among R5 HIV-1 Isolates From Individuals With Severe Immunodeficiency. *J Acquir Immune Defic Syndr*. Mar 01 2016;71(3):e79-82. doi:10.1097/QAI.0000000000000873
52. Jessen Krut J, Mellberg T, Price RW, et al. Biomarker evidence of axonal injury in neuroasymptomatic HIV-1 patients. *PLoS One*. 2014;9(2):e88591. doi:10.1371/journal.pone.0088591
53. Gisslén M, Fuchs D, Svennerholm B, et al. Cerebrospinal fluid viral load, intrathecal immunoactivation, and cerebrospinal fluid monocytic cell count in HIV-1 infection. *J Acquir Immune Defic Syndr*. Aug 01 1999;21(4):271-6. doi:10.1097/00126334-199908010-00003
54. Mellgren A, Antinori A, Cinque P, et al. Cerebrospinal fluid HIV-1 infection usually responds well to antiretroviral treatment. *Antivir Ther*. 2005;10(6):701-7.
55. Edén A, Fuchs D, Hagberg L, et al. HIV-1 viral escape in cerebrospinal fluid of subjects on suppressive antiretroviral treatment. *J Infect Dis*. Dec 15 2010;202(12):1819-25. doi:10.1086/657342
56. Gisslén M, Hunt PW. Antiretroviral Treatment of Acute HIV Infection Normalizes Levels of Cerebrospinal Fluid Markers of Central Nervous System (CNS) Inflammation: A Consequence of a Reduced CNS Reservoir? *J Infect Dis*. 11 06 2019;220(12):1867-1869. doi:10.1093/infdis/jiz031
57. Noyan K, Nguyen S, Betts MR, et al. Human Immunodeficiency Virus Type-1 Elite Controllers Maintain Low Co-Expression of Inhibitory Receptors on CD4+ T Cells. *Front Immunol*. 2018;9:19. doi:10.3389/fimmu.2018.00019
58. Akusjärvi SS, Ambikan AT, Krishnan S, et al. Integrative proteo-transcriptomic and immunophenotyping signatures of HIV-1 elite control phenotype: A cross-talk between glycolysis and HIF signaling. *iScience*. Jan 21 2022;25(1):103607. doi:10.1016/j.isci.2021.103607
59. Ceña-Diez R, Singh K, Spetz AL, et al. Novel Naturally Occurring Dipeptides and Single-Stranded Oligonucleotide Act as Entry Inhibitors and Exhibit a Strong Synergistic Anti-HIV-1 Profile. *Infect Dis Ther*. Jun 2022;11(3):1103-1116. doi:10.1007/s40121-022-00626-8
60. Neogi U, Siddik AB, Kalaghatgi P, et al. Recent increased identification and transmission of HIV-1 unique recombinant forms in Sweden. *Sci Rep*. 07 25 2017;7(1):6371. doi:10.1038/s41598-017-06860-2
61. Mellgren Å, Eriksson LE, Reinius M, et al. Longitudinal trends and determinants of patient-reported side effects on ART-a Swedish national registry study. *PLoS One*. 2020;15(12):e0242710. doi:10.1371/journal.pone.0242710

For peer review only

For peer review only - http://bmjopen.bmj.com/site/about/guidelines.xhtml

62. Tyrberg E, Edén A, Eriksen J, et al. Higher plasma drug levels in elderly people living with HIV treated with darunavir. *PLoS One*. 2021;16(2):e0246171. doi:10.1371/journal.pone.0246171
63. Carlsson-Lalloo E, Rusner M, Berg M, et al. People living with HIV in Sweden report high levels of sexual satisfaction in a registry-based cohort study. *AIDS Care*. 05 2022;34(5):559-567. doi:10.1080/09540121.2021.1909698
64. Carlsson-Lalloo E, Berg M, Rusner M, et al. Sexual satisfaction and its associations with patient-reported outcomes in a cohort of women living with human immunodeficiency virus in Sweden. *Int J STD AIDS*. 07 2022;33(8):751-760. doi:10.1177/09564624221100056
65. Carlander C, Wagner P, Yilmaz A, et al. Employment by HIV status, mode of HIV transmission and migrant status: a nation-wide population-based study. *AIDS (London, England)*. Jan 1 2021;35(1):115-123. doi:10.1097/qad.0000000000002724
66. Regioner SKo. Certifieringsnivå. 24th August, 2022. Accessed 24th August, 2022. https://skr.se/kvalitetsregister/hittaregister/certifieringsnivaer/certifieringsniva1.43960.html

For peer review only - http://bmjopen.bmj.com/site/about/guidelines.xhtml

632 **Table 1**

633 **Description of the InfCareHIV cohort, August 2022**

	In active care	Not active	All
**All**	8 436	4 593	13 029
**Sex**			
Female	3 322 (39.4)	1 042 (22.7)	4 364 (33.5)
**Current age**			
median (IQR)	50 (42-59)		
**Birth region**			
Sweden	2 820 (33.4)	1 708 (37.2)	4 528 (34.7)
Western Europe except Sweden	392 (4.6)	450 (9.8)	842 (6.5)
Eastern Europe and Central Asia	532 (6.3)	301 (6.5)	833 (6.4)
Asia and Pacific	853 (10.1)	191 (4.2)	1 044 (8.0)
Middle East and North Africa	238 (2.8)	74 (1.6)	312 (2.4)
Sub-Saharan Africa	3 069 (36.4)	1 006 (21.9)	4 075 (31.3)
Latin America and the Caribbean	370 (4.4)	179 (3.9)	549 (4.2)
North America	39 (0.5)	57 (1.2)	96 (0.7)
Missing	123 (1.5)	627 (13.6)	750 (5.8)
**Mode of HIV transmission**			
Heterosexual	4 270 (50.6)	1 544 (33.6)	5 814 (44.6)
Men who have sex with men/bisexual	2 657 (31.5)	1 707 (37.2)	4 364 (33.5)
Intravenous drug use	353 (4.2)	740 (16.1)	1 093 (8.4)
Mother to child	254 (3.0)	23 (0.5)	277 (2.1)
Blood products	116 (1.4)	118 (2.6)	234 (1.8)
Unknown/other	608 (7.2)	238 (5.2)	846 (6.5)
Missing	178 (2.1)	223 (4.9)	401 (3.1)
**Year of HIV diagnosis**			
1983-1996	1 384 (16.4)	2 482 (54.0)	3 866 (29.7)
1997-2006	2 209 (26.2)	867 (18.8)	3 076 (23.6)
2007-2016	4 367	971	4 438

For peer review only - http://bmjopen.bmj.com/site/about/guidelines.xhtml

	(41.1)	(21.1)	(34.1)
2017-2022	1 196 (14.2)	124 (2.7)	1 320 (10.1)
Missing	180 (2.1)	149 (3.2)	329 (2.5)
**Level of immunosuppression**			
Nadir CD4 cell count, median (IQR)	230 (122-370)	165 (40-330)	218 (90-359)
Missing nadir CD4 cell count, n (%)	26 (0.3)	449 (9.8)	475 (3.6)
Last registered CD4 cell count, median (IQR)	620 (460-813)	330 (93-560)	549 (340-750)
Missing last CD4 cell count, n (%)	27 (0.3)	449 (9.8)	476 (3.6)
**Antiretroviral therapy**			
On ART	8 305 (98.4)	-	-
Treated ≥6m with HIV-RNA<50 copies/mL	7 719 (94.6)	-	-
Treated ≥6m with HIV-RNA<200 copies/mL	8 267 (98.0)	-	-
Seven most common treatment combinations of those on ART (n=8 305)			
abacavir+lamivudine+dolutegravir	1 650 (19.9)	-	-
emtricitabine+tenofovir(TDF)+dolutegravir	1 256 (15.1)	-	-
lamivudine+dolutegravir	802 (9.7)	-	-
emtricitabine+tenofovir (TAF)+bictegravir	719 (8.7)	-	-
emtricitabine+tenofovir(TDF)+efavirenz	590 (7.1)	-	-
emtricitabin+tenofovir(TAF)+dolutegravir	574 (6.9)	-	-
emtricitabine+tenofovir(TAF)+rilpivirine	449 (5.4)	-	-
**Reason for not being in active care**			
Dead	-	2 575 (56.1)	-
Emigrated	-	1 584 (34.5)	-
Lost to follow-up	-	150 (3.3)	-
Reason not stated	-	284 (6.2)	-

Data are numbers and percentages (%) unless otherwise stated. Percentages do not always add up to hundred due to rounding. Birth regions according to UNAIDS definitions.

For peer review only - http://bmjopen.bmj.com/site/about/guidelines.xhtml

Abbreviations: ART: antiretroviral therapy, TAF: tenofovir alafenamide, TDF: tenofovir disoproxil fumarate

Figure 1. Clinical decision support system with results from self-reported health questionnaire below the graph.

Red symbolises low ratings that need attention, yellow medium ratings, and green contentment. Kroppslig hälsa; physical health, Psykiskt välmående; psychological health, Sexualitet; sexual health, Biverkningar; side effects, Delaktig i planering; patient participation in planning of care, Antal missade doser; missed doses of antiretroviral therapy, Rökning; smoking.

For peer review only

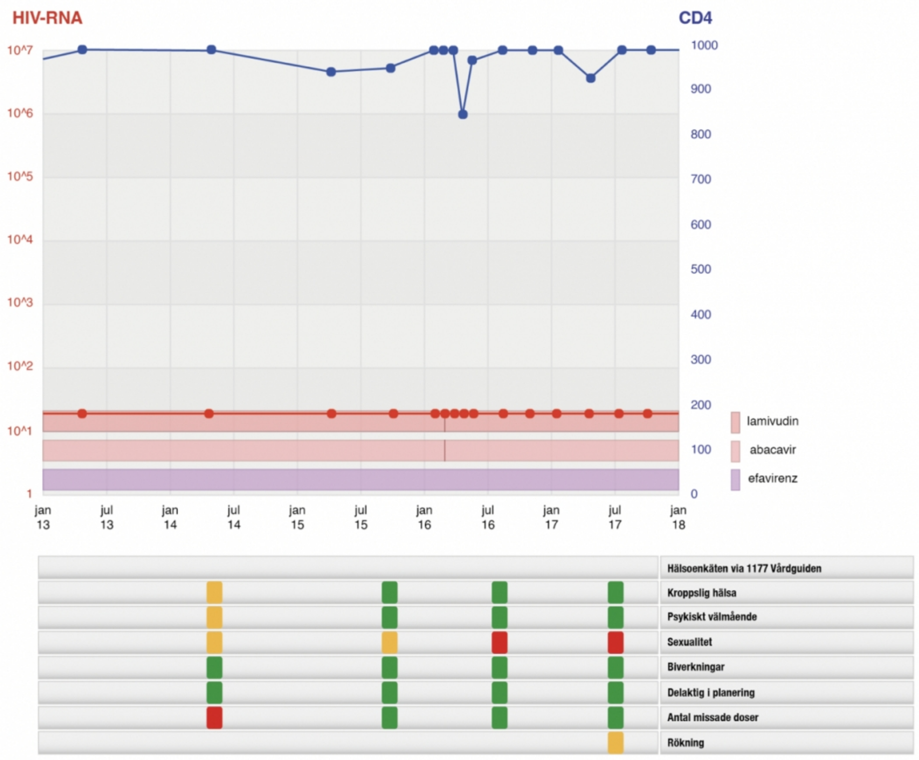

Clinical decision support system with results from self-reported health questionnaire below the graph.

246x198mm (144 x 144 DPI)

Supplementary Table 1. Summary of variables included in InfCareHIV

**Demographics**	
general	age, sex at birth, gender identity, ethnicity, country of birth
HIV-specific	mode of HIV-transmission, first ever positive HIV-serology, first positive HIV-serology in Sweden, suspected country of HIV transmission, last negative HIV-test, confirmed primary HIV infection
**Laboratory results**	
HIV-specific	HIV-1, HIV-2
	CD4+ count (absolute, %), CD8+ count (absolute, %), CD4/CD8 ratio, nadir CD4 count
	HIV-RNA copies /mL (plasma, CSF, sperm)
	genotypes, HIV sequences, elite-controllers
	HLA-B5701-allele
Other laboratory results	hepatitis A, B, C and D serologies, HBV-DNA, HCV-RNA
	syphilis serology
	SARS-CoV-2 (PCR)
Physical measurements	Weight, blood pressure etc.
**Treatment**	
Antiretroviral therapy	Start and stop dates, regimen (including dose and mode of administration), reason for change
Selected other medications	PCP-prophylaxis, Hepatitis C treatment etc.
**AIDS-diagnoses**	date and type of AIDS-diagnosis
**Co-morbidities**	diabetes type 1 and 2, hypertension, kidney failure, ischemic heart disease etc.
**Pregnancies**	dates of pregnancy, date of delivery etc.
**Death**	date and reason of death
**Health Questionnaire** *PROMs & PREMs*	Satisfaction with physical, psychological, and sexual health. Adherence to ART. Side effects. Smoking-habits. Satisfaction and involvement of care.

AIDS: acquired immunodeficiency syndrome, ART: antiretroviral therapy, CSF: cerebrospinal fluid, HBV: hepatis B virus, HCV: hepatitis C virus, HIV: human immunodeficiency virus, PCP: pneumocystis carinii prophylaxis, PROM: patient-reported outcome measures, PREM: patient-reported experience measures

For peer review only

Health survey Quality registry InfCareHIV

Personal number:
Birth year:
Name:

Please tick the appropriate box for each statement

1. How satisfied are you with your physical health?

Very unsatisfied	Unsatisfied	Rather unsatisfied	Rather satisfied	Satisfied	Very satisfied
☐	☐	☐	☐	☐	☐

2. How satisfied are you with your psychological wellbeing?

Very unsatisfied	Unsatisfied	Rather unsatisfied	Rather satisfied	Satisfied	Very satisfied
☐	☐	☐	☐	☐	☐

3. How satisfied are you with your sexual life (regardless if you have sex with a partner or on your own)?

Very unsatisfied	Unsatisfied	Rather unsatisfied	Rather satisfied	Satisfied	Very satisfied
☐	☐	☐	☐	☐	☐

4a. Are you currently taking HIV medication?

Yes ☐ à Go to question 4b. No ☐ à Go to question 5.

4b. Do you experience any side effects?

Yes ☐ à Go to question 4c. No ☐ à Go to question 4d.

4c. To what extent are you troubled by medical side effects?

Very troubled	Troubled	Rather troubled	Not very troubled	Not at all troubled
☐	☐	☐	☐	☐

4d. How many doses have you missed the last week?

0	1-2	3 or mote doses
☐	☐	☐

5. Do you smoke?

Never	Quitted	Yes
☐	☐	☐

6. Do you feel involved in the planning and realization of your HIV care and treatment?

Never	Seldom	Sometimes	Always
☐	☐	☐	☐

7. How satisfied are you with the quality of care provided at your HIV clinic?

Very unsatisfied	Unsatisfied	Rather unsatisfied	Rather satisfied	Satisfied	Very satisfied
☐	☐	☐	☐	☐	☐

Thank you for your participation!

For peer review only

BMJ Open

Cohort profile: InfCareHIV, a prospective registry-based cohort study of people with diagnosed HIV in Sweden

Journal:	*BMJ Open*
Manuscript ID	bmjopen-2022-069688.R1
Article Type:	Cohort profile
Date Submitted by the Author:	16-Feb-2023
Complete List of Authors:	Carlander, Christina; Karolinska University Hospital, Department of Infectious Diseases; Karolinska Institute, Department of Medicine Huddinge<br>Brännström, Johanna; Karolinska Institutet, Department of Medicine Huddinge; Södersjukhuset, Department of Infectious Diseases/Venhälsan<br>Mansson, Fredrik; Infectious Diseases Research Unit, Department of Clinical Sciences, Lund University<br>Elvstam, Olof; Lund University, Department of Translational Medicine; Växjö Central Hospital, Department of Infectious Diseases<br>Albinsson, Pernilla; Karolinska University Hospital<br>Blom, Simon; HIV Sweden<br>Mattsson, Lena; Karolinska University Hospital<br>Hovmöller, Sanne; Sunderby Hospital, Department of Infectious Diseases<br>Norrgren, Hans; Lund University Faculty of Science<br>Mellgren, Aasa; Sahlgrenska University Hospital, Department of Infectious Diseases<br>Svedhem, Veronica; Karolinska University Hospital, Department of Infectious Diseases; Karolinska Institutet, Department of Medicine Huddinge<br>Gisslén, Magnus; University of Gothenburg Sahlgrenska Academy, Department of Infectious Diseases, Institute of Biomedicine; Sahlgrenska University Hospital, Department of Infectious Diseases, Region Västra Götaland<br>Sonnerborg, Anders; Division of Infectious Diseases, Department of Medicine Huddinge, Karolinska Institutet and Division of Clinical Microbiology, Department of Laboratory Medicine, Karolinska Institute
**Primary Subject Heading**:	HIV/AIDS
Secondary Subject Heading:	Epidemiology, Infectious diseases, Patient-centred medicine
Keywords:	HIV & AIDS < INFECTIOUS DISEASES, Epidemiology < INFECTIOUS DISEASES, PREVENTIVE MEDICINE

For peer review only - http://bmjopen.bmj.com/site/about/guidelines.xhtml

SCHOLARONE™
Manuscripts

I, the Submitting Author has the right to grant and does grant on behalf of all authors of the Work (as defined in the below author licence), an exclusive licence and/or a non-exclusive licence for contributions from authors who are: i) UK Crown employees; ii) where BMJ has agreed a CC-BY licence shall apply, and/or iii) in accordance with the terms applicable for US Federal Government officers or employees acting as part of their official duties; on a worldwide, perpetual, irrevocable, royalty-free basis to BMJ Publishing Group Ltd ("BMJ") its licensees and where the relevant Journal is co-owned by BMJ to the co-owners of the Journal, to publish the Work in this journal and any other BMJ products and to exploit all rights, as set out in our licence.

The Submitting Author accepts and understands that any supply made under these terms is made by BMJ to the Submitting Author unless you are acting as an employee on behalf of your employer or a postgraduate student of an affiliated institution which is paying any applicable article publishing charge ("APC") for Open Access articles. Where the Submitting Author wishes to make the Work available on an Open Access basis (and intends to pay the relevant APC), the terms of reuse of such Open Access shall be governed by a Creative Commons licence – details of these licences and which Creative Commons licence will apply to this Work are set out in our licence referred to above.

Other than as permitted in any relevant BMJ Author's Self Archiving Policies, I confirm this Work has not been accepted for publication elsewhere, is not being considered for publication elsewhere and does not duplicate material already published. I confirm all authors consent to publication of this Work and authorise the granting of this licence.

For peer review only

BMJ Open Cohort profile

Title: Cohort profile: InfCareHIV, a prospective registry-based cohort study of people with diagnosed HIV in Sweden

Authors Christina Carlander[1,2], Johanna Brännström[2,3], Fredrik Månsson[4], Olof Elvstam[4,5], Pernilla Albinsson[2], Simon Blom[6], Lena Mattsson[1], Sanne Hovmöller[7], Hans Norrgren[8], Åsa Mellgren[9,10], Veronica Svedhem[1,2], Magnus Gisslén[9,10], Anders Sönnerborg[1,2]

Author affiliations

[1]Department of Infectious Diseases, Karolinska University Hospital, Stockholm, Sweden

[2]Division of Infectious Diseases, Department of Medicine Huddinge, Karolinska Institutet, Stockholm, Sweden

[3]Department of Infectious Diseases/Venhälsan, Södersjukhuset, Stockholm, Sweden

[4]Department of Translational Medicine, Lund University, Malmö, Sweden

[5]Department of Infectious Diseases, Växjö Central Hospital, Växjö, Sweden

[6]Ombudsman, HIV-Sweden, Stockholm, Sweden

[7]Department of Infectious Diseases, Sunderby Hospital, Luleå

[8]Department of Clinical Sciences Lund, Lund University, Lund, Sweden

[9]Department of Infectious Diseases, Institute of Biomedicine, Sahlgrenska Academy, University of Gothenburg

[10]Department of Infectious Diseases, Region Västra Götaland, Sahlgrenska University Hospital, Gothenburg, Sweden

Corresponding Author

Christina Carlander, MD, PhD, christina.carlander@ki.se

For peer review only

Abstract

Purpose The Swedish InfCareHIV cohort was established in 2003 to ensure equal and effective care of people living with HIV (PLHIV) and enable long-term follow-up. InfCareHIV functions equally as a decision support system as a quality registry, ensuring up-to-date data reported in real time.

Participants InfCareHIV includes data on >99% of all people with diagnosed HIV in Sweden and up to now 13,029 have been included in the cohort. InfCareHIV includes data on HIV-related biomarkers and antiretroviral therapies (ART) and also on demographics, patient-reported outcome measures and patient-reported experience measures.

Findings to date: Sweden was in 2015 the first country to reach the UNAIDS/WHO's 90-90-90 goals. Late diagnosis of HIV infection was identified as a key problem in the Swedish HIV-epidemic, and low-level HIV viremia while on ART associated to all-cause mortality. Increased HIV RNA load in the cerebrospinal fluid despite suppression of the plasma viral load was found in 5% of PLHIV, a phenomenon referred to as "CSF viral escape". Dolutegravir-based treatment in PLHIV with pre-existing NRTI-mutations was non-inferior to protease inhibitor-based regimens. An increase of transmitted drug resistance was observed in the InfCareHIV cohort. Lower efficacy for protease inhibitors was not due to lower adherence to treatment. Incidence of type 2 diabetes and insulin resistance was high in the aging HIV population. Despite ART, the risk of infection-related cancer as well as lung cancer was increased in PLHIV compared to HIV-negative. PLHIV were less likely successfully treated for cervical precancer and more likely to have human papillomavirus types not included in current HPV vaccines. Self-reported sexual satisfaction in PLHIV is improving and was higher in women than men.

Future plans InfCareHIV provides a unique base to study and further improve long-term treatment outcomes, comorbidity management and health-related quality of life in people with HIV in Sweden.

Strengths and limitations

- Complete nation-wide inclusion of all diagnosed people with HIV
- High accuracy, completeness, consistency, and validity of data
- Ability to link to other nation-wide health care registers
- Includes patient-reported outcomes
- Lacking information on socioeconomic data

For peer review only - http://bmjopen.bmj.com/site/about/guidelines.xhtml

Introduction

With access to antiretroviral therapy (ART) the overall life expectancy of people living with HIV (PLHIV) is close to that of HIV-seronegative people, as shown in a study including European and North American HIV cohorts (1). However, studies from the Swedish InfCareHIV cohort show that after a 15-year follow-up period, successfully treated PLHIV in Sweden were three times more likely to die when compared with HIV-seronegative controls (2). The InfCareHIV cohort is heterogenous, including a large proportion of migrants from 108 countries on all continents, diverse modes of HIV transmission and socioeconomic backgrounds and is gender balanced. The *pol* sequence of the virus is characterized at diagnosis regarding drug resistance mutations and subtype. All known HIV-1 subtypes, many recombinants as well as unique recombinant forms and HIV-2 are represented. Pre-treatment HIV drug resistance is a critical aspect that requires global collaborative studies and to minimize its effect, retention in care and optimal adherence to treatment is essential (3).

The Swedish HIV cohort is aging and faced with increasing risk of age-related comorbidities. Also, chronic HIV-related inflammation, legacy of pre-treatment immunodeficiency and injury, and past and present ART may add to the development of comorbidity (4, 5). Additionally, half of PLHIV in Sweden are still late presenters, defined as CD4 count<350 and/or AIDS at time of diagnosis, which has well-known consequences for prognosis and transmission (6, 7). Despite undetectable plasma viral levels, health-related quality of life (HRQoL) is shown to be poorer compared with HIV-seronegative people, in a study from the United Kingdom (8, 9). This article describes the prospective Swedish HIV cohort, InfCareHIV, including all diagnosed people living with HIV in Sweden, with data dating back to the earliest cases in the 1980ies.

Cohort description

Study population

The InfCareHIV cohort was established in 2003 in the two largest cities in Sweden, Stockholm, and Gothenburg. These two cities care for about half of all PLHIV in Sweden. The other Swedish clinics joined thereafter and since 2008 the cohort has

For peer review only - http://bmjopen.bmj.com/site/about/guidelines.xhtml

had complete national coverage, including all 29 clinical HIV centres in Sweden. To date (August 2022), 13 029 PLHIV have been included of whom 8,436 are currently in active care (Table 1, Figure 1). In total 42 PLHIV have been diagnosed with HIV-2 of whom 28 are in active care. In the beginning of the HIV epidemic the majority of PLHIV were born in Sweden, most of whom were MSM (57.5%) or transmitted through intravenous drug use (26.9%) (Supplementary Table 1). The number of migrants with HIV, in particular from sub-Saharan Africa (SSA) and Asia, increased gradually, as did the proportion of women and heterosexually transmitted individuals (Supplementary Table 1). A study performed between 2009 and 2012 found 58% of newly diagnosed in Sweden to be late presenters (CD4 < 350 cells/μL or AIDS) and 38% to have an advanced infection (<200 cells/μL or AIDS). Ten years later the proportion of late diagnosis remains high (63% and 42% with CD4<350 and <200 respectively) (6, 7).

Of those in active care (August 2022) 39% are women and 1.2% are children below 18 years of age. A majority was born outside Sweden (67%), most commonly in SSA (36%). About half (51%) have stated heterosexual mode of transmission, about a third are men who have sex with men or bisexual men (31%). Only 4% have stated intravenous drug use as mode of transmission while 3% were mother-to-child transmissions, 1.4% through blood products and for 9.6% transmission mode is unknown or information is missing (Table 1). Almost all are on ART (98%), and of them almost everybody (95%) reaches the treatment goal of HIV-RNA<50 copies/mL (98% HIV-RNA <200 copies/mL) in snapshot analysis after at least six months of treatment (10). Of the 4,483 people no longer on follow-up in InfCareHIV, 56% are deceased, 34% have emigrated and 3% are lost-to follow-up. InCareHIV also includes undocumented migrants who often end-up among the 3% that are lost-to follow-up (Table 1). Retrospective data from before the cohort was established as nation-wide in 2008 has been backlogged, including PLHIV deceased before 2008.

Coverage and validation of the study population

The national coverage is nearly 100% as all 29 clinical centres that attend PLHIV are included in InfCareHIV, and more than 99% of all diagnosed PLHIV who are living in Sweden are included. This number has been repeatedly validated on an aggregated level, most recently in 2019, against the number of HIV-diagnoses reported to the

For peer review only - http://bmjopen.bmj.com/site/about/guidelines.xhtml

Public Health Agency of Sweden, a report that is mandatory by Swedish law. The validations found that data regarding HIV diagnoses were even more accurate in InfCareHIV than in the Public Health Agencies records. When transferring between clinics the person keeps their individual InfCareHIV identity number with no data lost.

Cohort variables

Variables collected and manually registered by a health professional at enrolment include sex at birth, gender identity (added 2021), country of birth, mode of HIV transmission, date of any last negative HIV-test and first positive HIV-test (in Sweden and if relevant abroad), any AIDS diagnoses (for example pneumocystis pneumonia, esophageal candidiasis or tuberculosis), confirmed primary HIV infection and suspected country of HIV transmission (Supplementary Table 2). Data are collected/updated at each follow-up visit; manually or automatically depending on the type of data and the HIV centre. Data include ART start and stop dates, ART regimen (including doses and mode of administration) and the reason for any change of drug regimens, prophylaxis of opportunistic infections and selected co-medications. Serological and virological data on co-infection with hepatitis C and B virus, weight, date and type of AIDS-defining events, date and cause of death are also included. HIV-RNA (plasma and cerebrospinal fluid [CSF]) and HIV drug resistance (including viral sequences) results, described as both mutations and predicted phenotypic sensitivity, as well as CD4+ and CD8+ T-cell counts and CD4/CD8 ratios are either automatically or manually included depending on the HIV centre. HIV-1 subtype and HLA B-5701-allele are registered (Supplementary Table 2). Pregnancies and following deliveries are also registered. Socioeconomic data such as civil status, education level or income is not included in InfCareHIV.

Validation of cohort variables

Validation of data is performed regularly on national level using a Data Quality Index, with the possibility to validate also on clinic and individual level. Data Quality Index for InfCareHIV is currently at 4.9 on a 0-5 scale. Data are also validated through the Distributed Data Management tool which enables data extraction for data sharing and automatically conducts quality assurance checks that signal any incorrect values that may then be manually corrected. This tool is derived and modified from a

For peer review only

collaboration with EuroCoord, the European Network of HIV/AIDS Cohort studies to coordinate at European and International Level Clinical Research on HIV/AIDS (11).

Ethics

At time of entering HIV care, PLHIV are informed about the registry, after which they can either give verbal consent or opt out. Participants always have the right to exit InfCareHIV, although this has been very uncommon. Participants can request an extract on their data from the registry, free of charge, in accordance with the European General Data Protection Regulation (GDPR 2016/679) and the Swedish Data Protection Act (2018:218). This cohort study has been approved by the Regional Ethical committees in Sweden (Dnr 532-11, Dnr 2018/11-31/2, Dnr 2022-02897-02).

Patient and public involvement

A representative from a Swedish organization for PLHIV (www.hiv-sverige.se) is co-opted to the InfCareHIV steering committee. A webpage (www.infcarehiv.se) contains information for both PLHIV, professionals involved in HIV care, researchers, and the public. Statistics and treatment results of the individual clinical centres are published openly in an annual report and on the web page, more on that below.

Decision support system

The mainstay of InfCareHIV is the clinical decision support system (Figure 1). Data on HIV-RNA levels (plasma and CSF), CD4 cell counts (absolute and percentage), drug resistance, and previous and current ART are presented through a graphical system. In addition, the legend below the graph presents data regarding date of HIV diagnosis, date of first ART and names of the HIV-team consisting of doctor, nurse, and counsellor. Periods of pregnancy are also shown in the graph. All other information is easily accessible in the system. Also, since all viral (*pol*) sequences are stored in the database, reanalysis can easily be performed when new drugs or new information about resistance appear. Thus, data generated in clinical care are automatically "reused" for research and development. The graph gives the clinician a relevant visual summary that is easier to grasp than the medical records, especially in treatment experienced PLHIV with a long history of different ART. Meanwhile the graph is also a pedagogical instrument during patient consultation, facilitating patient-

For peer review only - http://bmjopen.bmj.com/site/about/guidelines.xhtml

centered care, making it easy to illustrate for example the consequences of good or poor treatment adherence. HRQoL is also illustrated in the graph (Figure 2). Finally, the graph is used when transferring PLHIV between clinic centres, for expert-consultancy and at multi-disciplinary team conferences.

Quality Registry

An important feature of InfCareHIV is to function as a National Quality Registry. The aim of a quality registry is to develop and ensure quality of care systematically and continuously. This allows for comparisons on HIV care at a national, regional, and clinical centre level and has led to increased equality of care on national level with diminishing differences in treatment results in Sweden's HIV centres. We believe it is most likely that this contributed to that Sweden in 2015 was the first country to reach the United Nations Programme on HIV/AIDS/World Health Organization´s 90-90-90 goal with 90% diagnosed, 90% of them on ART and 90% of them with viral suppression (10). In concordance with WHO/UNAIDS updated goal, we are now measuring the 95-95-95 goal and have also added 95 goals for HRQoL.

Health-related quality of life

Systematical quantification of patient-reported outcomes (PROs) will assist the improvement of medical care and HRQoL of PLHIV. In 2011, a 9-item self-reported Health Questionnaire (HQ) was integrated to the registry to be answered annually by PLHIV either via a website or using a computerized or paper version at the outpatient clinic (Supplementary Table 3) (12). The questionnaire was developed together with representatives from PLHIV patient organizations. The HQ assesses patient-reported outcome measures (PROMs) regarding physical, psychological, and sexual health, self-reported ART adherence, experience of side effects and patient-reported experience measures (PREMs) regarding involvement and satisfaction with care. A question on smoking habits was added in 2017. The questionnaire is electronically available in Swedish and English and in paper version in the eight most common languages in the InfCareHIV cohort. Illiterate patients are offered help by a nurse/interpreter. The results are presented in the Decision support system (Figure 2) and used at the patient´s routine clinical follow-up visit. The HQ enhances patient-centered HIV care by focusing the consultation on the patients current needs. The red signal symbolises low ratings *(very unsatisfied/unsatisfied),* that need attention,

For peer review only - http://bmjopen.bmj.com/site/about/guidelines.xhtml

yellow medium ratings *(rather unsatisfied/rather satisfied)*, and green contentment *(satisfied/very satified),* (Supplementary Table 3). There is a standardized clinical manual for the health personnel with suggestions on how to manage yellow and red alerts (for example screening for depression and ask about intimate partner violence if psychological well-being gets a yellow or red alert). The HQ is validated by content validity and test-retest reliability and has also been evaluated as a tool for longitudinal follow up of trends in PROs (12-14).Our fourth 95 goal is that 95% of all PLHIV should perform the health questionnaire and that 95% of PLHIV should have an ART regimen without experience of side-effects. The results from the HQ are also used in research, more on that below.

HIV-related stigma and discrimination are major obstacles for reaching good HRQoL. In close relation to InfCareHIV we developed a 12-item HIV stigma scale, a short version of the commonly used 40-item HIV Stigma Scale by Berger et al (15). It is a valid and reliable instrument for the measurement of enacted, anticipated, and internalized stigma and crucial for mapping trends in the prevalence of HIV-related stigma and tracking the effectiveness of stigma-reducing interventions (15, 16).

Biobank

In the major clinical centres blood plasma/serum/liquor samples are collected, and in selected cases also peripheral blood mononuclear cells, HIV isolates, CSF and CSF cells in biobanks. These biobanks are separate from the cohort but linking between the cohort data and the biobanks can be performed (after ethical permission) for translational studies.

Selected findings from the past ten years to date

Studies on late presentation

In a national prospective study, using InfCareHIV linked to patient study forms, Brännström et al. found late diagnosis of HIV infection to be a key problem in the Swedish HIV-1 epidemic, where more than half of the patients were diagnosed late, but the majority could have been diagnosed earlier with a more efficient health care system (6, 17, 18). Most of the patients experienced barriers to HIV testing, but less so if the HIV-test was offered through screening programs or by a health care professional rather than having to be self-initiated (19, 20). Results from the study

For peer review only - http://bmjopen.bmj.com/site/about/guidelines.xhtml

have been adopted by the Public Health Agency of Sweden and have had implications for the Swedish governmental National strategy against HIV/AIDS and other infectious diseases (21).

Studies on co-morbidity and mortality

Elvstam et al. reported an association between low-level HIV viremia while on ART and all-cause mortality (22), and has further explored the effects of detectable viremia during ART by linking InfCareHIV to National Health registries and analyzing stored biobank samples (23-27). Malmström et al. studied risk of cancer by HIV status in Sweden for three decades, finding that PLHIV have a remaining increased risk of infection-related cancer despite ART, while lung cancer was the only non-infection-related cancer increased in PLHIV (28). By linking InfCareHIV to the National Cervical Cancer Screening registry, Carlander et al. showed that PLHIV are less likely to have successful treatment of cervical precancer and more likely to have human papillomavirus (HPV) types not included in current HPV vaccines (29-32). These results were used when the national cervical-cancer-screening recommendation was updated in 2022 (33). Brännström et al. showed that the incidence of type 2 diabetes and insulin resistance is high in the aging HIV population, where comorbidities are common and associated whereas there was no association found for HIV related factors (34). Möller et al. showed that well-treated PLHIV are not at higher odds of severe COVID-19 compared with HIV-negative people after controlling for age and comorbidity (35).

Studies on HIV infection of the central nervous system

Gisslén et al. have used InfCareHIV in a substantial number of studies exploring CSF viral load and other biomarkers in different settings of untreated and treated HIV (36-54). Viral load is normally 1 log lower in CSF than in plasma in untreated HIV (55), but CSF exceeds plasma HIV RNA in approximately 15% of patients, with considerable variations between different disease stages (36). CNS infection is generally well controlled by systemic suppressive ART (56), although in approximately 5% the HIV RNA load was increased in the CSF despite suppression of the plasma viral load, a phenomenon referred to as "CSF viral escape" (41). This phenomenon is most often transient, not associated with any symptoms and comparable to plasma viral blips (57) which occur in about the same frequency (58). Several studies have indicated that that a stable, permanent infection of cells in the

For peer review only - http://bmjopen.bmj.com/site/about/guidelines.xhtml

CNS is established later than in systemic viral reservoirs (40), which has implications when exploring HIV eradication strategies (59).

Studies on Elite Controllers

Sönnerborg et al have extensively studied, since the 1990-thies, long-term nonprogressors and elite controllers (EC), selected from the InfCareHIV cohort. Specific immunological (60) and metabolic features have been described (61). Also, a naturally occurring dipeptide was found to be enhanced among EC and to possess antiretroviral properties, acting as both an entry inhibitor and an RT-inhibitor (62).

Studies on drug resistance mutations

Sönnerborg et al have also studied HIV drug resistance, both transmitted and acquired, and the molecular HIV epidemiology. Among several key findings, an increase of transmitted drug resistance in the InfCareHIV cohort (3) was reported. A pronounced HIV-1 subtype heterogeneity, including all known subtypes, many circulating recombinant forms and unique recombinant forms was also described (63). Sörstedt et al. studied the effect of dolutegravir-based treatment in PLHIV with pre-existing NRTI-mutations and found a non-inferior effect compared to protease inhibitor based regimens (64) and has also explored viral blips during ART (58).

Studies on patient-reported outcomes and experiences

Svedhem et al. validated the Health Questionnaire and demonstrated that self-reported adherence in the HQ was correlated to viral suppression and described determinants of optimal ART-adherence in the cohort (12). Svedhem et al. have shown that the assessment of PROMs is an important tool to ensure the long-term adherence to treatment, improvement in quality of life and evaluate side-effects on HIV treatment (12, 14). PROMs have also successfully been used in virological molecular modeling showing that lower affinity for protease inhibitors to HIV-1C protease do not depend on lower adherence to treatment among people infected with subtype C (13). Tyberg et al. compared plasma drug levels of ART, potential drug-drug interactions and side-effects in PWH aged ≥ 65 years of age, with controls ≤ 49 years of age, and found differences in drug concentrations and reported side effects between groups (65). Mellgren et al demonstrated that the experience of side-effects of ART declined significantly in the cohort during 2011-2017 and that experiences of side effects were diverse and associated with both self-reported physical and psychological health (14). Two studies on self-reported sexuality by Mellgren et al. found that self-reported sexual satisfaction in PLHIV improved annually and that women were more satisfied

For peer review only

For peer review only - http://bmjopen.bmj.com/site/about/guidelines.xhtml

with their sexual life compared with men (66, 67). In women living with HIV, satisfaction with sexual life was associated with self-reported psychological health and experiences of side-effects. Carlander et al. showed that over the past twenty years access to employment has increased in PLHIV although remaining lower compared to HIV-negative, even after controlling for migrant and socioeconomic status (68).

International collaborations

InfCareHIV has collaborated or collaborates with several international HIV cohorts such as EuroCoord, EuroSIDA, RESPOND, PENTA, COHERE, CASCADE, CHAIN, NEAT, EuResist, EuCare, UCSF CSF Cohort, and CARE.

Future plans

InfCareHIV provides a unique base to continue study long-term treatment, comorbidities and HRQoL of people with HIV in Sweden. Half of all PLHIV is still diagnosed late and studies that can help improve HIV-testing guidelines and HIV awareness are essential. Attempts are made to develop more precise bioinformatics tools for assessment of time of infection and the undiagnosed population. Pre-treatment HIV drug resistance is a critical aspect that requires global collaboration and long-term follow-up. Despite most PLHIV reaching the treatment goal of undetectable viral level, patient reported health is still poorer than for HIV-negative people and studies on how to improve HRQoL in PLHIV and minimize stigma should be prioritized. With our aging cohort, studies on comorbidity becomes more important and specially to assess what comorbidities are associated with normal aging and what comorbidities may be associated with ART or HIV-induced chronic inflammation. Multiomics using samples and clinical information is presently evaluated to identify biomarkers for prediction of comorbidities, to characterize the evolution of central nervous system (CNS) inflammation and injury (45) and for future selection of patients in HIV cure attempts.

Strengths and limitations

The main strength of the InfCareHIV cohort is that it includes all diagnosed PLHIV in Sweden. The accuracy, completeness, consistency and validity is very high and consequently InfCareHIV has the highest certification level among Swedish Health

For peer review only

Quality registries according to the Swedish Association of Local Authorities and Regions (69). The personal identity number given to all Swedish residents enables linkage of data to any Swedish population or health registry after appropriate application, providing opportunities to answer a wide range of research questions. Also, the automatic transfer of key laboratory data, including viral sequences, and the possibility of linking cohort data and biobanks allow easy reuse of information obtained in clinical care for research purposes. Some variables in InfCareHIV (e.g., comorbidity, weight and self-reported smoking status that has been added in later years) lacks in coverage which currently limits their use in research but linking to other health registries for the collection of this data is then a possibility. The aim is for all PLHIV to be invited to answer the self-reported health questionnaire annually to facilitate patient centered care and improve HRQOL, although currently we reach only about 34%, and it is a prioritized matter to improve this number.

Collaboration We encourage collaborations with HIV cohorts from other countries. Data can be made available upon reasonable request and after ethical approval.

Further details

Author affiliations

[1]Department of Infectious Diseases, Karolinska University Hospital, Stockholm, Sweden
[2]Division of Infectious Diseases, Department of Medicine Huddinge, Karolinska Institutet, Stockholm, Sweden
[3]Department of Infectious Diseases/Venhälsan, Södersjukhuset, Stockholm, Sweden
[4]Department of Translational Medicine, Lund University, Malmö, Sweden
[5]Department of Infectious Diseases, Växjö Central Hospital, Växjö, Sweden
[6]Ombudsman, HIV-Sweden, Stockholm, Sweden
[7]Department of Infectious Diseases, Sunderby Hospital, Luleå
[8]Department of Clinical Sciences Lund, Lund University, Lund, Sweden
[9]Department of Infectious Diseases, Institute of Biomedicine, Sahlgrenska Academy, University of Gothenburg
[10]Department of Infectious Diseases, Region Västra Götaland, Sahlgrenska University Hospital, Gothenburg, Sweden

Acknowledgments First of all, the authors would like to thank all people living with HIV in Sweden enrolled in the cohort. We also thank all clinical centres that work daily with the inclusion and update of data in InfCareHIV.

Contributors Contributors JB, FM, OE, PA, SB, LM, SH, HN, ÅM, VS, MG, and AS planned and designed the cohort profile description. CC drafted the manuscript.

For peer review only - http://bmjopen.bmj.com/site/about/guidelines.xhtml

JB, FM, OE, PA, SB, LM, SH, HN, ÅM, VS, MG, and AS critically revised the manuscript and approved the final version.

Funding No funding to report for this paper.

Competing interests CC has received lecture, moderator, and advisory board fees from GSK/ViiV, Gilead Sciences and MSD and an unrestricted Nordic Fellowship Grant from Gilead Sciences Nordic. JB has received lecture and advisory board fees from GSK/ViiV and Gilead. FM has received lecture and advisory board fees from GSK/ViiV, Astra Zeneca and Gilead Sciences. OE has received a grant to his institution from Pfizer and honoraria as speaker from Gilead Sciences. HN has received advisory board fees from Gilead and Abbvie. ÅM has received lecture and advisory board fees from GSK/ViiV, Gilead Sciences and Pfizer. MG has received research grants from Gilead Sciences and Janssen-Cilag and honoraria as speaker, DSMB committee member and/or scientific advisor from Amgen, AstraZeneca Biogen, Bristol-Myers Squibb, Gilead Sciences, GlaxoSmithKline/ViiV, Janssen-Cilag, MSD, Novocure, Novo Nordic, Pfizer and Sanofi. AS has received research grants from Gilead Sciences and honoraria as speaker, DSMB committee member and/or scientific advisor from AstraZeneca, Bristol-Myers Squibb, Gilead Sciences, GlaxoSmithKline/ViiV, and MSD. All other authors declare no competing interests

Patient consent for publication Not applicable.

Ethics approval This cohort study has been approved by the Regional Ethical committees in Sweden (Dnr 532-11, Dnr 2018/11-31/2, Dnr 2022-02897-02).

Provenance and peer review Not commissioned, externally peer reviewed.

Data availability statement Data can be made available upon reasonable request and after ethical approval.

References

1. Survival of HIV-positive patients starting antiretroviral therapy between 1996 and 2013: a collaborative analysis of cohort studies. The Lancet HIV. 2017;4(8):e349-e56.
2. de Coninck Z, Hussain-Alkhateeb L, Bratt G, et al. Non-AIDS Mortality Is Higher Among Successfully Treated People Living with HIV Compared with Matched HIV-Negative Control Persons: A 15-Year Follow-Up Cohort Study in Sweden. AIDS Patient Care STDS. 2018;32(8):297-305.
3. Andersson E, Ambikan A, Brännström J, et al. High-throughput sequencing reveals a high prevalence of pretreatment HIV-1 drug resistance in Sweden. AIDS (London, England). 2021;35(2):227-34.

For peer review only

For peer review only - http://bmjopen.bmj.com/site/about/guidelines.xhtml

4. Schnittman SR, Deitchman AN, Beck-Engeser G, et al. Abnormal Levels of Some Biomarkers of Immune Activation Despite Very Early Treatment of Human Immunodeficiency Virus. The Journal of infectious diseases. 2021;223(9):1621-30.
5. Gabuzda D, Jamieson BD, Collman RG, et al. Pathogenesis of Aging and Age-related Comorbidities in People with HIV: Highlights from the HIV ACTION Workshop. Pathogens & immunity. 2020;5(1):143-74.
6. Brannstrom J, Svedhem Johansson V, Marrone G, et al. Deficiencies in the health care system contribute to a high rate of late HIV diagnosis in Sweden. HIV medicine. 2016;17(6):425-35.
7. InfCareHIV [Online] 2022. Available from: https://infcarehiv.se (accessed 24 August 2022).
8. Lazarus JV, Safreed-Harmon K, Barton SE et al. Beyond viral suppression of HIV - the new quality of life frontier. BMC Med. 2016;14(1):94.
9. Miners A, Phillips A, Kreif N, et al. Health-related quality-of-life of people with HIV in the era of combination antiretroviral treatment: a cross-sectional comparison with the general population. Lancet HIV. 2014;1(1):e32-40.
10. Gisslén M, Svedhem V, Lindborg L, et al. Sweden, the first country to achieve the Joint United Nations Programme on HIV/AIDS (UNAIDS)/World Health Organization (WHO) 90-90-90 continuum of HIV care targets. HIV medicine. 2017;18(4):305-7.
11. Commission E. European network of HIV/AIDS cohort studies to coordinate at European and International Level Clinical Research on HIV/AIDS [Online] 2015. Available from: https://cordis.europa.eu/project/id/260694 (accessed 24 August 2022).
12. Marrone G, Mellgren Å, Eriksson LE, et al. High Concordance between Self-Reported Adherence, Treatment Outcome and Satisfaction with Care Using a Nine-Item Health Questionnaire in InfCareHIV. PloS one. 2016;11(6):e0156916.
13. Häggblom A, Svedhem V, Singh K, et al. Virological failure in patients with HIV-1 subtype C receiving antiretroviral therapy: an analysis of a prospective national cohort in Sweden. Lancet HIV. 2016;3(4):e166-74.
14. Mellgren Å, Eriksson LE, Reinius M, et al. Longitudinal trends and determinants of patient-reported side effects on ART-a Swedish national registry study. PLoS One. 2020;15(12):e0242710.
15. Reinius M, Wettergren L, Wiklander M, et al. Development of a 12-item short version of the HIV stigma scale. Health Qual Life Outcomes. 2017;15(1):115.
16. Reinius M, Wiklander M, Wettergren L, et al. The Relationship Between Stigma and Health-Related Quality of Life in People Living with HIV Who Have Full Access to Antiretroviral Treatment: An Assessment of Earnshaw and Chaudoir's HIV Stigma Framework Using Empirical Data. AIDS Behav. 2018;22(12):3795-806.
17. Brännström J, Svedhem V, Marrone G, et al. Symptomatic Patients without Epidemiological Indicators of HIV Have a High Risk of Missed Diagnosis: A Multi-Centre Cross Sectional Study. PLoS One. 2016;11(9):e0162503.
18. Brännström J, Sönnerborg A, Svedhem V, et al. A high rate of HIV-1 acquisition post immigration among migrants in Sweden determined by a CD4 T-cell decline trajectory model. HIV Med. 2017;18(9):677-84.
19. Wiklander M, Brännström J, Svedhem V, et al. Development and psychometric testing of a barriers to HIV testing scale among individuals with HIV

For peer review only - http://bmjopen.bmj.com/site/about/guidelines.xhtml

infection in Sweden; The Barriers to HIV testing scale-Karolinska version. Health Qual Life Outcomes. 2015;13:185.

20. George-Svahn L, Eriksson LE, Wiklander M, et al. Barriers to HIV testing as reported by individuals newly diagnosed with HIV infection in Sweden. AIDS Care. 2021;33(10):1286-95.

21. Sweden GOo. National strategy against HIV/AIDS and certain other transmittable diseases [Online] 2017. Available from: https://www.regeringen.se/4ad776/globalassets/regeringen/dokument/socialdepartementet/fokhalsa-och-sjukvard/nationell-strategi-mot-hiv-aids-och-vissa-andra-smittsamma-sjukdomar.pdf (accessed 14 February 2023).

22. Elvstam O, Marrone G, Medstrand P, et al. All-Cause Mortality and Serious Non-AIDS Events in Adults With Low-level Human Immunodeficiency Virus Viremia During Combination Antiretroviral Therapy: Results From a Swedish Nationwide Observational Study. Clin Infect Dis. 2021;72(12):2079-86.

23. Elvstam O, Medstrand P, Yilmaz A, et al. Virological failure and all-cause mortality in HIV-positive adults with low-level viremia during antiretroviral treatment. PLoS One. 2017;12(7):e0180761.

24. Elvstam O, Medstrand P, Jansson M, et al. Is low-level HIV-1 viraemia associated with elevated levels of markers of immune activation, coagulation and cardiovascular disease? HIV Med. 2019;20(9):571-80.

25. Elvstam O, Marrone G, Medstrand P, et al. Associations Between Plasma Human Immunodeficiency Virus (HIV) Ribonucleic Acid Levels and Incidence of Invasive Cancer in People With HIV After Initiation of Combination Antiretroviral Therapy. Open Forum Infect Dis. 2021;8(6):ofab131.

26. Brattgård H, Björkman P, Nowak P, et al. Factors associated with low-level viraemia in people with HIV starting antiretroviral therapy: A Swedish observational study. PLoS One. 2022;17(5):e0268540.

27. Elvstam O, Marrone G, Engström G, et al. Associations between HIV viremia during antiretroviral therapy and cardiovascular disease. AIDS. 2022.

28. Malmström S, Wagner P, Yilmaz A, et al. Failure to restore CD4+ cell count associated with infection-related and noninfection-related cancer. AIDS (London, England). 2022;36(3):447-57.

29. Carlander C, Wagner P, van Beirs A, et al. Suppressive antiretroviral therapy associates with effective treatment of high-grade cervical intraepithelial neoplasia. AIDS (London, England). 2018;32(11):1475-84.

30. Carlander C, Wagner P, Svedhem V, et al. Impact of immunosuppression and region of birth on risk of cervical intraepithelial neoplasia among migrants living with HIV in Sweden. Int J Cancer. 2016.

31. Carlander C, Lagheden C, Eklund C, et al. HPV Types in Cervical Precancer by HIV Status and Birth Region: A Population-Based Register Study. Cancer epidemiology, biomarkers & prevention : a publication of the American Association for Cancer Research, cosponsored by the American Society of Preventive Oncology. 2020;29(12):2662-8.

32. Carlander C, Lagheden C, Eklund C, et al. Nonvaccine human papillomavirus genotype common in women with HIV failing cervical precancer treatment. AIDS (London, England). 2021;35(14):2367-74.

33. Cancercentrum. Prevention of cervical cancer. National health guidelines [Online] 2022. Available from: https://cancercentrum.se/globalassets/vara-uppdrag/prevention-tidig-upptackt/gynekologisk-

For peer review only

For peer review only - http://bmjopen.bmj.com/site/about/guidelines.xhtml

cellprovskontroll/vardprogram/nationellt-vardprogram-cervixcancerprevention.pdf. (accessed 14 February 2023).
34. Bratt G, Brännström J, Missalidis C, et al. Development of type 2 diabetes and insulin resistance in people with HIV infection: Prevalence, incidence and associated factors. PLoS One. 2021;16(6):e0254079.
35. Möller I, Gisslén M, Wagner P, et al. Clinical outcome in people with COVID-19 by HIV-status: a nationwide register study. CROI; Virtual2022.
36. Ulfhammer G, Edén A, Antinori A, et al. Cerebrospinal Fluid viral load across the spectrum of untreated HIV-1 infection: a cross-sectional multi-center study. Clin Infect Dis. 2021.
37. Hagberg L, Price RW, Zetterberg H, et al. Herpes zoster in HIV-1 infection: The role of CSF pleocytosis in secondary CSF escape and discordance. PLoS One. 2020;15(7):e0236162.
38. Joseph SB, Kincer LP, Bowman NM, Evans C, et al. Human Immunodeficiency Virus Type 1 RNA Detected in the Central Nervous System (CNS) After Years of Suppressive Antiretroviral Therapy Can Originate from a Replicating CNS Reservoir or Clonally Expanded Cells. Clin Infect Dis. 2019;69(8):1345-52.
39. Ulfhammer G, Edén A, Mellgren Å, et al. Persistent central nervous system immune activation following more than 10 years of effective HIV antiretroviral treatment. AIDS. 2018;32(15):2171-8.
40. Burbelo PD, Price RW, Hagberg L, et al. Anti-Human Immunodeficiency Virus Antibodies in the Cerebrospinal Fluid: Evidence of Early Treatment Impact on Central Nervous System Reservoir? J Infect Dis. 2018;217(7):1024-32.
41. Edén A, Nilsson S, Hagberg L, et al. Asymptomatic Cerebrospinal Fluid HIV-1 Viral Blips and Viral Escape During Antiretroviral Therapy: A Longitudinal Study. J Infect Dis. 2016;214(12):1822-5.
42. Yilmaz A, Yiannoutsos CT, Fuchs D, et al. Cerebrospinal fluid neopterin decay characteristics after initiation of antiretroviral therapy. J Neuroinflammation. 2013;10:62.
43. Anesten B, Zetterberg H, Nilsson S, et al. Effect of antiretroviral treatment on blood-brain barrier integrity in HIV-1 infection. BMC Neurol. 2021;21(1):494.
44. Yilmaz A, Mellgren Å, Fuchs D, et al. Switching from a regimen containing abacavir/lamivudine or emtricitabine/tenofovir disoproxil fumarate to emtricitabine/tenofovir alafenamide fumarate does not affect central nervous system HIV-1 infection. Infect Dis (Lond). 2019;51(11-12):838-46.
45. Gisslen M, Keating SM, Spudich S, et al. Compartmentalization of cerebrospinal fluid inflammation across the spectrum of untreated HIV-1 infection, central nervous system injury and viral suppression. PLoS One. 2021;16(5):e0250987.
46. Hermansson L, Yilmaz A, Axelsson M, et al. Cerebrospinal fluid levels of glial marker YKL-40 strongly associated with axonal injury in HIV infection. J Neuroinflammation. 2019;16(1):16.
47. Yilmaz A, Fuchs D, Price RW, et al. Cerebrospinal Fluid Concentrations of the Synaptic Marker Neurogranin in Neuro-HIV and Other Neurological Disorders. Curr HIV/AIDS Rep. 2019;16(1):76-81.

For peer review only - http://bmjopen.bmj.com/site/about/guidelines.xhtml

48. Gisslén M, Heslegrave A, Veleva E, et al. CSF concentrations of soluble TREM2 as a marker of microglial activation in HIV-1 infection. Neurol Neuroimmunol Neuroinflamm. 2019;6(1):e512.
49. Anesten B, Yilmaz A, Hagberg L, et al. Blood-brain barrier integrity, intrathecal immunoactivation, and neuronal injury in HIV. Neurol Neuroimmunol Neuroinflamm. 2016;3(6):e300.
50. Ahlgren E, Hagberg L, Fuchs D, et al. Association between Plasma Homocysteine Levels and Neuronal Injury in HIV Infection. PLoS One. 2016;11(7):e0158973.
51. Krut JJ, Price RW, Zetterberg H, Fuchs D, et al. No support for premature central nervous system aging in HIV-1 when measured by cerebrospinal fluid phosphorylated tau (p-tau). Virulence. 2017;8(5):599-604.
52. Gisslén M, Price RW, Andreasson U, et al. Plasma Concentration of the Neurofilament Light Protein (NFL) is a Biomarker of CNS Injury in HIV Infection: A Cross-Sectional Study. EBioMedicine. 2016;3:135-40.
53. Karlsson U, Repits J, Antonsson L, et al. Reduced Baseline Sensitivity to Maraviroc Inhibition Among R5 HIV-1 Isolates From Individuals With Severe Immunodeficiency. J Acquir Immune Defic Syndr. 2016;71(3):e79-82.
54. Jessen Krut J, Mellberg T, Price RW, et al. Biomarker evidence of axonal injury in neuroasymptomatic HIV-1 patients. PLoS One. 2014;9(2):e88591.
55. Gisslén M, Fuchs D, Svennerholm B, et al. Cerebrospinal fluid viral load, intrathecal immunoactivation, and cerebrospinal fluid monocytic cell count in HIV-1 infection. J Acquir Immune Defic Syndr. 1999;21(4):271-6.
56. Mellgren A, Antinori A, Cinque P, et al. Cerebrospinal fluid HIV-1 infection usually responds well to antiretroviral treatment. Antivir Ther. 2005;10(6):701-7.
57. Edén A, Fuchs D, Hagberg L, et al. HIV-1 viral escape in cerebrospinal fluid of subjects on suppressive antiretroviral treatment. J Infect Dis. 2010;202(12):1819-25.
58. Sörstedt E, Nilsson S, Blaxhult A, et al. Viral blips during suppressive antiretroviral treatment are associated with high baseline HIV-1 RNA levels. BMC Infect Dis. 2016;16:305.
59. Gisslén M, Hunt PW. Antiretroviral Treatment of Acute HIV Infection Normalizes Levels of Cerebrospinal Fluid Markers of Central Nervous System (CNS) Inflammation: A Consequence of a Reduced CNS Reservoir? J Infect Dis. 2019;220(12):1867-9.
60. Noyan K, Nguyen S, Betts MR, et al. Human Immunodeficiency Virus Type-1 Elite Controllers Maintain Low Co-Expression of Inhibitory Receptors on CD4+ T Cells. Front Immunol. 2018;9:19.
61. Akusjärvi SS, Ambikan AT, Krishnan S, et al. Integrative proteo-transcriptomic and immunophenotyping signatures of HIV-1 elite control phenotype: A cross-talk between glycolysis and HIF signaling. iScience. 2022;25(1):103607.
62. Ceña-Diez R, Singh K, Spetz AL, et al. Novel Naturally Occurring Dipeptides and Single-Stranded Oligonucleotide Act as Entry Inhibitors and Exhibit a Strong Synergistic Anti-HIV-1 Profile. Infect Dis Ther. 2022;11(3):1103-16.
63. Neogi U, Siddik AB, Kalaghatgi P, et al. Recent increased identification and transmission of HIV-1 unique recombinant forms in Sweden. Sci Rep. 2017;7(1):6371.
64. Sörstedt E, Carlander C, Flamholc L, et al. Effect of dolutegravir in combination with Nucleoside Reverse Transcriptase Inhibitors (NRTIs) on people

For peer review only - http://bmjopen.bmj.com/site/about/guidelines.xhtml

living with HIV who have pre-existing NRTI mutations. Int J Antimicrob Agents. 2018;51(5):733-8.
65. Tyrberg E, Edén A, Eriksen J, et al. Higher plasma drug levels in elderly people living with HIV treated with darunavir. PLoS One. 2021;16(2):e0246171.
66. Carlsson-Lalloo E, Rusner M, Berg M, et al. People living with HIV in Sweden report high levels of sexual satisfaction in a registry-based cohort study. AIDS Care. 2022;34(5):559-67.
67. Carlsson-Lalloo E, Berg M, Rusner M, et al. Sexual satisfaction and its associations with patient-reported outcomes in a cohort of women living with human immunodeficiency virus in Sweden. Int J STD AIDS. 2022;33(8):751-60.
68. Carlander C, Wagner P, Yilmaz A, et al. Employment by HIV status, mode of HIV transmission and migrant status: a nation-wide population-based study. AIDS (London, England). 2021;35(1):115-23.
69. Regioner SKo. Certifieringsnivå [Online] 2022. Available from: https://skr.se/kvalitetsregister/hittaregister/certifieringsnivaer/certifieringsniva1.43960.html (accessed 24 August 2022).

For peer review only

Table 1

Description of the InfCareHIV cohort, August 2022

	In active care	Not active	All
**All**	8 436	4 593	13 029
**Sex**			
Female	3 322 (39.4)	1 042 (22.7)	4 364 (33.5)
**Birth region**			
Sweden	2 820 (33.4)	1 708 (37.2)	4 528 (34.7)
Western Europe except Sweden	392 (4.6)	450 (9.8)	842 (6.5)
Eastern Europe and Central Asia	532 (6.3)	301 (6.5)	833 (6.4)
Asia and Pacific	853 (10.1)	191 (4.2)	1 044 (8.0)
Middle East and North Africa	238 (2.8)	74 (1.6)	312 (2.4)
Sub-Saharan Africa	3 069 (36.4)	1 006 (21.9)	4 075 (31.3)
Latin America and the Caribbean	370 (4.4)	179 (3.9)	549 (4.2)
North America	39 (0.5)	57 (1.2)	96 (0.7)
Missing	123 (1.5)	627 (13.6)	750 (5.8)
**Mode of HIV transmission**			
Heterosexual	4 270 (50.6)	1 544 (33.6)	5 814 (44.6)
Men who have sex with men/bisexual	2 657 (31.5)	1 707 (37.2)	4 364 (33.5)
Intravenous drug use	353 (4.2)	740 (16.1)	1 093 (8.4)
Mother to child	254 (3.0)	23 (0.5)	277 (2.1)
Blood products	116 (1.4)	118 (2.6)	234 (1.8)
Unknown/other	608 (7.2)	238 (5.2)	846 (6.5)
Missing	178 (2.1)	223 (4.9)	401 (3.1)
**Year of HIV diagnosis**			
1979-1996	1 384 (16.4)	2 482 (54.0)	3 866 (29.7)
1997-2006	2 209 (26.2)	867 (18.8)	3 076 (23.6)
2007-2016	4 367 (41.1)	971 (21.1)	4 438(34.1)
2017-2022	1 196 (14.2)	124 (2.7)	1 320 (10.1)
Missing	180 (2.1)	149 (3.2)	329 (2.5)
**Level of immunosuppression**			
Nadir CD4 cell count, median (IQR)	230 (122-370)	165 (40-330)	218 (90-359)
Missing nadir CD4 cell count, n (%)	26 (0.3)	449 (9.8)	475 (3.6)
Last registered CD4 cell count, median (IQR)	620 (460-813)	330 (93-560)	549 (340-750)
**Antiretroviral therapy**			
On ART	8 305 (98.4)	-	-
Treated ≥6m with HIV-RNA<50 copies/mL	7 719 (94.6)	-	-
Treated ≥6m with HIV-RNA<200 copies/mL	8 267 (98.0)	-	-
Seven most common treatment combinations of those on ART (n=8 305)			
abacavir+lamivudine+dolutegravir	1 650 (19.9)	-	-
emtricitabine+tenofovir(TDF)+dolutegravir	1 256 (15.1)	-	-
lamivudine+dolutegravir	802 (9.7)	-	-
emtricitabine+tenofovir (TAF)+bictegravir	719 (8.7)	-	-
emtricitabine+tenofovir(TDF)+efavirenz	590 (7.1)	-	-
emtricitabin+tenofovir(TAF)+dolutegravir	574 (6.9)	-	-
emtricitabine+tenofovir(TAF)+rilpivirine	449 (5.4)	-	-
**Reason for not being in active care**			
Dead	-	2 575 (56.1)	-
Emigrated	-	1 584 (34.5)	-
Lost to follow-up	-	150 (3.3)	-
Reason not stated	-	284 (6.2)	-

For peer review only

For peer review only - http://bmjopen.bmj.com/site/about/guidelines.xhtml

Data are numbers and percentages (%) unless otherwise stated. Lab data (CD4 cell count and HIV-RNA viral loads) and data regarding antiretroviral therapy refer to latest available data unless otherwise stated. Individual data is generally updated at least every six months. PLHIV diagnosed <1983 were diagnosed retrospectively on biobanked blood once HIV-testing was introduced. Percentages do not always add up to hundred due to rounding. Birth regions according to UNAIDS definitions. Abbreviations: ART: antiretroviral therapy, TAF: tenofovir alafenamide, TDF: tenofovir disoproxil fumarate

Figure 1. Year of HIV diagnosis

Footnote

People with diagnosis <1983 were diagnosed retrospectively on biobanked blood once HIV-testing was introduced.

Figure 2. Clinical decision support system with results from self-reported health questionnaire below the graph

Footnote

Red symbolises low ratings that need attention (very unsatisfied/unsatisfied), yellow medium ratings (rather unsatisfied/rather satisfied), and green contentment (satisfied/very satisfied), Supplementary Table 3. Kroppslig hälsa; physical health, Psykiskt välmående; psychological health, Sexualitet; sexual health, Biverkningar; side effects, Delaktig i planering; patient participation in planning of care, Antal missade doser; missed doses of antiretroviral therapy, Rökning; smoking.

For peer review only

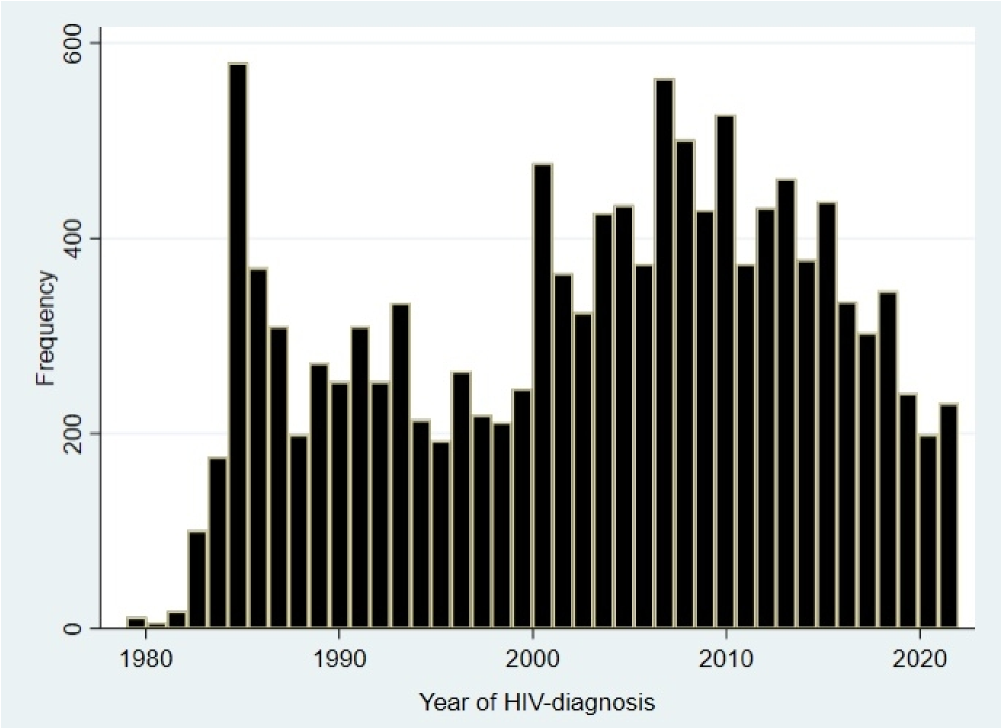

Figure 1. Year of HIV diagnosis
Footnote: People with diagnosis <1983 were diagnosed retrospectively on biobanked blood once HIV-testing was introduced.

121x88mm (144 x 144 DPI)

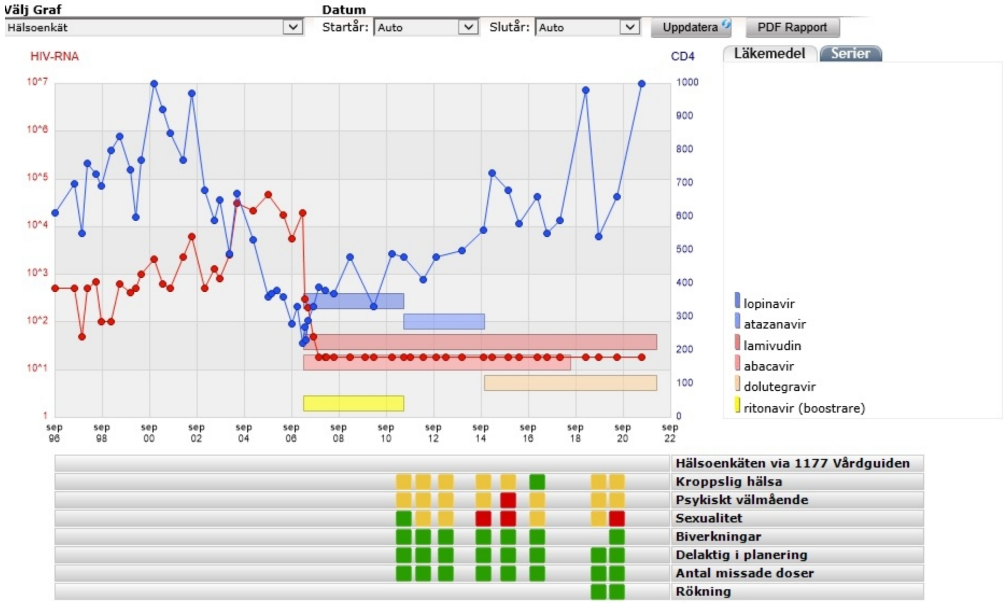

Figure 2. Clinical decision support system with results from self-reported health questionnaire below the graph

Footnote: Red symbolises low ratings that need attention (very unsatisfied/unsatisfied), yellow medium ratings (rather unsatisfied/rather satisfied), and green contentment (satisfied/very satisfied), Supplementary Table 3. Kroppslig hälsa; physical health, Psykiskt välmående; psychological health, Sexualitet; sexual health, Biverkningar; side effects, Delaktig i planering; patient participation in planning of care, Antal missade doser; missed doses of antiretroviral therapy, Rökning; smoking.

245x154mm (96 x 96 DPI)

Supplementary Table 1
Epidemiology of the InfCareHIV cohort

**Year of HIV diagnosis**	**<1987**	**1987-1996**	**1997-2006**	**2007-2016**	**2017-2022**	**unknown**	**All**
**All**	1264 (9.7)	2602 (20.0)	3076 (23.6)	4438 (34.1)	1320 (10.1)	324 (2.5)	13029
**Sex**							
Female	163 (12.9)	729 (28.0)	1225 (39.8)	1685 (38.0)	433 (33.1)	129 (42.3)	4364 (33.5)
**Birth region**							
Sweden	786 (62.2)	1153 (44.3)	1060 (34.5)	1131 (25.5)	362 (27.4)	36 (10.9)	4528 (34.7)
Western Europe except Sweden	128 (10.1)	225 (8.7)	193 (6.3)	233 (5.2)	53 (4.0)	10 (3.0)	842 (6.5)
Eastern Europe and Central Asia	22 (1.7)	60 (2.3)	169 (5.5)	391 (8.8)	152 (11.5)	39 (11.8)	833 (6.5)
Asia and Pacific	6 (0.5)	77 (3.0)	313 (10.2)	470 (10.6)	149 (11.3)	29 (8.8)	1044 (8.0)
Middle East and North Africa	6 (0.5)	33 (1.3)	53 (1.7)	149 (3.4)	63 (4.8)	8 (2.4)	312 (2.4)
Sub-Saharan Africa	26 (2.1)	632 (24.3)	1125 (36.6)	1775 (40.0)	421 (31.9)	96 (29.2)	4 075 (31.3)
Latin America and the Caribbean	19 (1.5)	92 (3.5)	115 (3.7)	228 (5.1)	85 (6.4)	10 (3.0)	549 (4.2)
North America	11 (0.9)	35 (1.3)	12 (4.4)	28 (0.6)	6 (0.5)	4 (1.2)	96 (0.7)
Missing	260 (20.6)	295 (11.3)	36 (1.2)	33 (0.7)	24 (2.2)	97 (29.5)	750 (5.8)
**Mode of HIV transmission**							
Heterosexual	68 (5.4)	1072 (41.2)	1758 (57.1)	2243 (50.5)	599 (45.4)	74 (22.5)	5814 (44.6)
Men who have sex with men/bisexual	727 (57.5)	1015 (34.0)	763 (24.8)	1369 (30.9)	443 (33.6)	47 (12.3)	4364 (33.5)
Intravenous drug use	340 (26.9)	268 (10.3)	240 (7.8)	201 (4.5)	35 (2.6)	9 (2.7)	1093 (8.4)
Mother to child	3 (0.2)	42 (1.6)	86 (2.8)	114 (2.6)	19 (1.4)	13 (3.9)	277 (2.1)
Blood products	104 (8.2)	49 (1.6)	37 (1.2)	34 (0.8)	8 (0.6)	2 (0.6)	234 (1.8)
Unknown/other	9 (0.7)	86 (3.3)	165 (5.3)	396 (8.9)	155 (11.7)	35 (10.6)	846 (6.5)
Missing	13 (1.0)	70 (2.7)	27 (0.9)	81 (1.8)	61 (4.6)	149 (45.3	401 (3.1)
**Level of immunosuppression**							
Nadir CD4 cell count, median (IQR)	90 (20-200)	150 (42-258)	204 (110-300)	278 (145-420)	300 (130-480)	385 (205-555)	218 (90-359)
Missing nadir CD4 cell count, n (%)	149 (11.8)	198 (7.6)	21 (0.7)	28 (0.6)	8 (0.6)	66 (20)	475 (3.6)

Data are numbers and percentages (%) unless otherwise stated. Percentages do not always add up to hundred due to rounding. Birth regions according to UNAIDS definitions.

For peer review only

Supplementary Table 2. Summary of variables included in InfCareHIV

**Demographics**	
general	age, sex at birth, gender identity, ethnicity, country of birth
HIV-specific	mode of HIV-transmission, first ever positive HIV-serology, first positive HIV-serology in Sweden, suspected country of HIV transmission, last negative HIV-test, confirmed primary HIV infection
**Laboratory results**	
HIV-specific	HIV-1, HIV-2
	CD4+ count (absolute, %), CD8+ count (absolute, %), CD4/CD8 ratio, nadir CD4 count
	HIV-RNA copies /mL (plasma, CSF, sperm)
	genotypes, HIV sequences, elite-controllers
	HLA-B5701-allele
Other laboratory results	hepatitis A, B, C and D serologies, HBV-DNA, HCV-RNA
	syphilis serology
	SARS-CoV-2 (PCR)
Physical measurements	Weight, blood pressure etc.
**Treatment**	
Antiretroviral therapy	Start and stop dates, regimen (including dose and mode of administration), reason for change
Selected other medications	PCP-prophylaxis, Hepatitis C treatment etc.
**AIDS-diagnoses**	date and type of AIDS-diagnosis
**Co-morbidities**	diabetes type 1 and 2, hypertension, kidney failure, ischemic heart disease etc.
**Pregnancies**	dates of pregnancy, date of delivery etc.
**Death**	date and reason of death
**Health Questionnaire** *PROMs & PREMs*	Satisfaction with physical, psychological, and sexual health. Adherence to ART. Side effects. Smoking-habits. Satisfaction and involvement of care.

AIDS: acquired immunodeficiency syndrome, ART: antiretroviral therapy, CSF: cerebrospinal fluid, HBV: hepatis B virus, HCV: hepatitis C virus, HIV: human immunodeficiency virus, PCP: pneumocystis carinii prophylaxis, PROM: patient-reported outcome measures, PREM: patient-reported experience measures

For peer review only

Health survey Quality registry InfCareHIV

Personal number:
Birth year:
Name:

Please tick the appropriate box for each statement

1. How satisfied are you with your physical health?

Very unsatisfied	Unsatisfied	Rather unsatisfied	Rather satisfied	Satisfied	Very satisfied
☐	☐	☐	☐	☐	☐

2. How satisfied are you with your psychological wellbeing?

Very unsatisfied	Unsatisfied	Rather unsatisfied	Rather satisfied	Satisfied	Very satisfied
☐	☐	☐	☐	☐	☐

3. How satisfied are you with your sexual life (regardless if you have sex with a partner or on your own)?

Very unsatisfied	Unsatisfied	Rather unsatisfied	Rather satisfied	Satisfied	Very satisfied
☐	☐	☐	☐	☐	☐

4a. Are you currently taking HIV medication?

Yes ☐ à Go to question 4b. No ☐ à Go to question 5.

4b. Do you experience any side effects?

Yes ☐ à Go to question 4c. No ☐ à Go to question 4d.

4c. To what extent are you troubled by medical side effects?

Very troubled	Troubled	Rather troubled	Not very troubled	Not at all troubled
☐	☐	☐	☐	☐

4d. How many doses have you missed the last week?

0	1-2	3 or mote doses
☐	☐	☐

5. Do you smoke?

Never	Quitted	Yes
☐	☐	☐

6. Do you feel involved in the planning and realization of your HIV care and treatment?

Never	Seldom	Sometimes	Always
☐	☐	☐	☐

7. How satisfied are you with the quality of care provided at your HIV clinic?

Very unsatisfied	Unsatisfied	Rather unsatisfied	Rather satisfied	Satisfied	Very satisfied
☐	☐	☐	☐	☐	☐

Thank you for your participation!

For peer review only
